# New information on paleopathologies in non-avian theropod dinosaurs: a case study on South American abelisaurids

**DOI:** 10.1186/s12862-023-02187-x

**Published:** 2024-01-31

**Authors:** Mattia A. Baiano, Ignacio A. Cerda, Filippo Bertozzo, Diego Pol

**Affiliations:** 1 School of Life Sciences, The Chinese University of Hong Kong, Shatin, Hong Kong SAR, China; 2 Consejo Nacional de Investigaciones Científicas y Técnicas (CONICET), Godoy Cruz 2290, 1425 Ciudad Autónoma de Buenos Aires, Argentina; 3 Area Laboratorio e Investigación, Museo Municipal ‘Ernesto Bachmann’, Dr Natali S/N, 8311 Villa El Chocon, Neuquén, Argentina; 4 Universidad Nacional de Río Negro (UNRN), Isidro Lobo 516, 8332 General Roca, Río Negro Argentina; 5 Instituto de Investigacion en Paleobiología y Geología (IIPG), Av. Roca 1242, 8332 General Roca, Río Negro Argentina; 6 Museo Carlos Ameghino, Belgrano 1700 (Paraje Pichi Ruca, Predio Marabunta), 8324 Cipolletti, Río Negro Argentina; 7 Operational Directorate Earth and History of Life, Royal Belgian Institute of Natural Sciences, Brussels, Belgium; 8 Museo Paleontológico Egidio Feruglio, Av. Fontana 140, 9100 Trelew, Chubut Argentina

**Keywords:** Spondyloarpthopathy, Congenital malformation, Radial fibrolamelar bone, Theropoda, Abelisauridae, Paleopathology, Paleohistology, CT-scan

## Abstract

Studies on pathological fossil bones have allowed improving the knowledge of physiology and ecology, and consequently the life history of extinct organisms. Among extinct vertebrates, non-avian dinosaurs have drawn attention in terms of pathological evidence, since a wide array of fossilized lesions and diseases were noticed in these ancient organisms. Here, we evaluate the pathological conditions observed in individuals of different brachyrostran (Theropoda, Abelisauridae) taxa, including *Aucasaurus garridoi*, *Elemgasem nubilus*, and *Quilmesaurus curriei*. For this, we use multiple methodological approaches such as histology and computed tomography, in addition to the macroscopic evaluation. The holotype of *Aucasaurus* shows several pathognomonic traits of a failure of the vertebral segmentation during development, causing the presence of two fused caudal vertebrae. The occurrence of this condition in *Aucasaurus* is the first case to be documented so far in non-tetanuran theropods. Regarding the holotype of *Elemgasem*, the histology of two fused vertebrae shows an intervertebral space between the centra, thus the fusion is limited to the distal rim of the articular surfaces. This pathology is here considered as spondyloarthropathy, the first evidence for a non-tetanuran theropod. The microstructural arrangement of the right tibia of *Quilmesaurus* shows a marked variation in a portion of the outer cortex, probably due to the presence of the radial fibrolamellar bone tissue. Although similar bone tissue is present in other extinct vertebrates and the cause of its formation is still debated, it could be a response to some kind of pathology. Among non-avian theropods, traumatic injuries are better represented than other maladies (e.g., infection, congenital or metabolic diseases, etc.). These pathologies are recovered mainly among large-sized theropods such as Abelisauridae, Allosauridae, Carcharodontosauridae, and Tyrannosauridae, and distributed principally among axial elements. Statistical tests on the distribution of injuries in these theropod clades show a strong association between taxa-pathologies, body regions-pathologies, and taxa-body regions, suggesting different life styles and behaviours may underlie the frequency of different injuries among theropod taxa.

**Supplementary Information:**

The online version contains supplementary material available at 10.1186/s12862-023-02187-x.

## Introduction

Studies on pathological fossil bones has allowed to know about physiology and ecology of extinct organism [1], providing a better knowledge about life history (e.g., [2–4]), inter- and intraspecific relationships [5–18], and behaviour (e.g., [19, 20]). Among extinct vertebrates, non-avian dinosaurs have drawn attention also in terms of pathological evidences, since different current diseases are noticed in these successful animals. In fact, the paleopathology record in non-avian dinosaurs includes fractures [2–4, 18, 21–25], amputations [14, 26], bite marks and scratches [5, 6, 11, 27, 28], cancer and tumor growth [29–32], developmental disorders [33–36] as well as different kinds of infections [2, 37–39]. Furthermore, the presence of some kind of paleopathology was indirectly inferred in theropod dinosaurs through anomalous footprints [40, 41]. However, some theropod groups were poorly explored under a paleopathological approach, and the presence of some diseases in these groups is still unknown. Taking into account Abelisauridae, the best known theropod group from the Late Cretaceous of Gondwana [42–45], paleopathological studies were carried out only on the Malagasy *Majungasaurus crenatissimus* [24, 26].

Here, we have identified three different pathologies in three specimens of the South American brachyrostran abelisaurids: *Aucasaurus garridoi*, *Elemgasem nubilus*, and *Quilmesaurus curriei*. In this work, we have carried out a macroscopic description of the pathological bones for each specimen and use the paleohistology and alternatively CT-scan to assess the modification of the internal microstructure and then to understand the response of the bones affected by maladies. So far, pathological specimens from South America were reported only among ornithopod, sauropodomorph, and tetanuran dinosaurs [31, 38, 46–54], but none for non-tetanuran theropods. Here we present the first study on paleopathologies for the clade Brachyrostra (Theropoda, Ceratosauria), and at the same time the third one for the clade Abelisauridae after the works on *Majungasaurus*. With this contribution, we also present the first occurrences of pathologies in non-tetanuran theropods from South America.

Finally, we have performed an exhaustive search in the literature to create a database of all pathologies recovered in non-avian theropods. We have analyzed the distribution of these diseases among different non-avian theropod groups and among different portions of the skeleton. Subsequently, these data were analyzed using statistical tests to understand if there is dependency among independent variables as a result of specific ecological interactions.

### Institutional abbreviations

AMNH, American Museum of Natural History, New York, USA; FMNH, Field Museum of Natural History, Chicago, Illinois, USA; MCF, Museo Carmen Funes, Plaza Huincul, Neuquén province, Argentina; MPCA, Museo Provincial Carlos Ameghino, Cipolletti, Río Negro province, Argentina; SMA, Sauriermuseum Aathal, Zürich, Switzerland; WDC, Wyoming Dinosaur Center, Warm Springs Ranch, Wyoming, USA.

## Materials and methods

In the past, the studies on pathological non-avian dinosaur specimens were generally carried out using only macroscopic evidences [14, 38, 50, 52, 55–59]. Nowadays, the utilization of alternative methodologies such as computed tomography (CT-scan) and paleohistology has complemented the gross morphological description to study several diseases in fossil specimens [23, 26, 31, 35, 60–62]. These techniques have allowed to know the internal arrangement of the pathological bones and how diseases affect the microstructure. Specifically, they have aided to recognize pathognomonic traits of different pathologies, useful when a differential diagnosis is proposed [23, 63]. Among non-avian dinosaurs, the paleohistology and CT-scan were used in pathological studies mainly for ornithischian [15, 25, 32, 60, 64–66] and sauropodomorph dinosaurs [48, 49, 51, 67–72], whereas they were poorly utilized for pathological theropod specimens [23, 26, 73, 74].

**Fig. 1:**
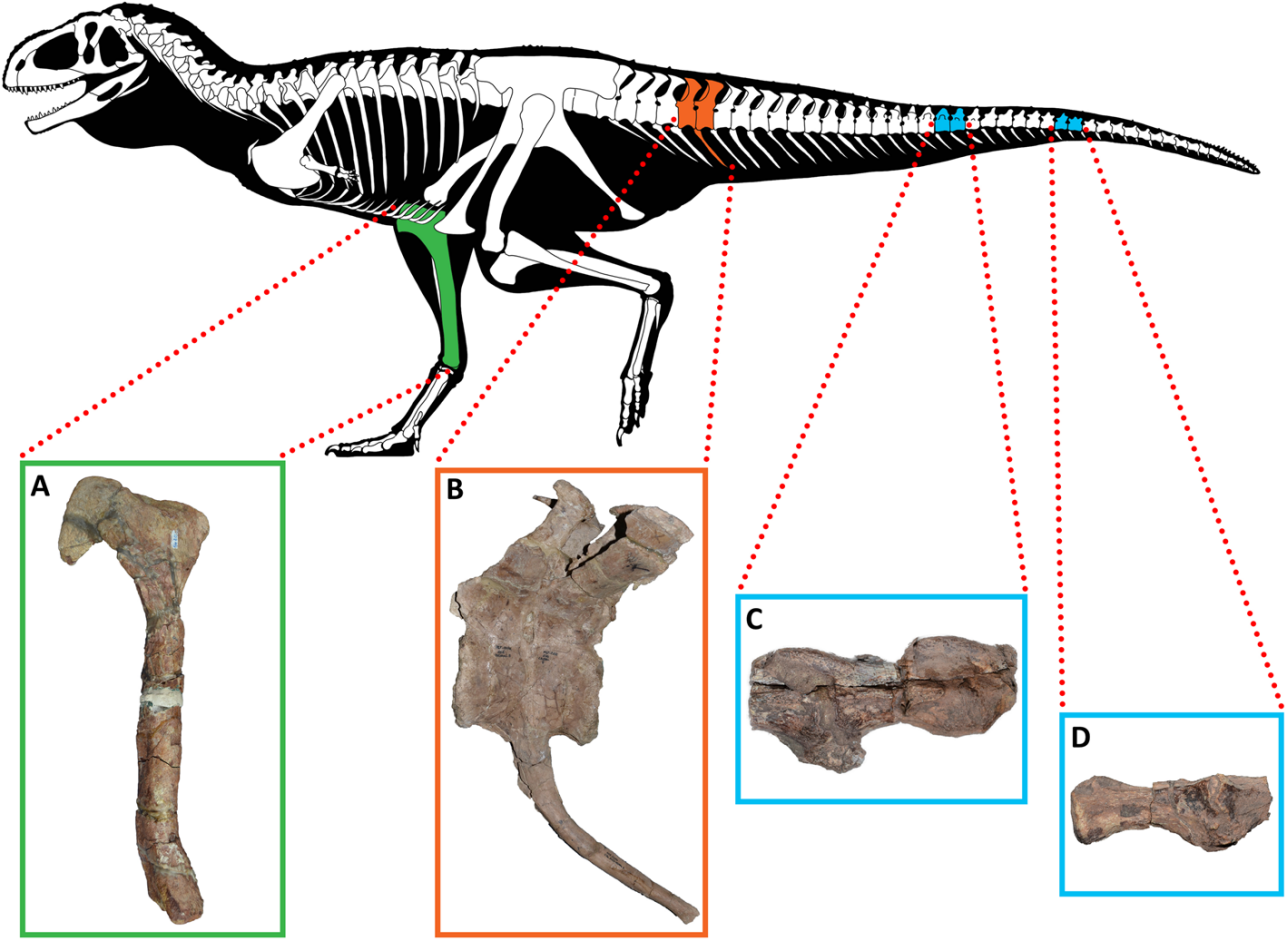
Elements of Patagonian abelisaurids affected by pathology. **A**, right tibia of *Quilmesaurus curriei* MPCA-PV-100 in medial view; **B**, 5th and 6th caudal vertebrae and 5th haemal arch of *Aucasaurus garridoi* MCF-PVPH-236, in left lateral view; **C** and **D**, middle and posterior caudal vertebrae of *Elemgasem nubilus* MCF-PVPH-380, in left lateral view. Artwork (silhouette and skeleton) by Alessio Ciaffi

The pathological abelisaurid specimens studied here are elements of the holotypes of *Aucasaurus garridoi* (MCF-PVPH-236) [75] and *Elemgasem nubilus* (MCF-PVPH-380) [45], housed in the Museo Municipal Carmen Funes [76], and *Quilmesaurus curriei* (MPCA-PV-100) [77], housed in the Museo Provincial Carlos Ameghino [78]. In particular, we have identified pathological conditions in the right tibia of *Quilmesaurus* (Fig. 1A), in the 5th and 6th caudal vertebrae and 5th haemal arch of *Aucasaurus* (Fig. 1B), and in three middle and two posterior caudal vertebrae and in two middle and a single posterior haemal arch of *Elemgasem* (Fig. 1C, D). To understand what kind of diseases affected those individuals, we have studied the pathological material under multiple approaches, proposing possible aetiologies. *In primis*, we described the external morphology of structures related with the pathology, and then we carried out histological analyses and computed tomography scans (CT-scans) to know the internal arrangement of the bone tissue. Detailed anatomical information of the bone sampled here are elsewhere provided [45, 77, 79].

To properly visualize the internal architecture of the pathological bones in *Aucasaurus garridoi* we performed a computed tomography scan (CT scan) to the 5th and 6th caudal vertebrae. The CT scan was performed using the scanner model Aquilion Lightnight 16/32, in the Sanatorio Plaza Huincul, at Plaza Huincul city (Neuquén Province, Argentina). The tomography was carried out taking into account the transverse, coronal, and parasagittal planes with the following settings: 120 kVp, 50 mA, and slices thickness of 5-mm. The slices were observed using the default software K-PACS produced by Ebit (ESAOTE).

The bone microstructure of a pair of fused caudal vertebrae of *Elemgasem nubilus* and of the right tibia of *Quilmesaurus curriei* was analyzed using bone histology. In the case of *Elemgasem nubilus,* sagittal and longitudinal sections were obtained from the area corresponding to the articulation of two fused mid-caudal centra. These sections include approximately the half of two consecutive centra. Whereas the longitudinal section comprises both left and right sides of the centra, the sagittal one only includes the ventral half. For the tibia of *Quilmesaurus curriei*, a single transversal section from the midshaft was analyzed. The pathological bone in the tibia was detected in a preliminary histological study of the holotype of *Quilmesaurus curriei* [80]. Histological sections were prepared at the petrographic laboratory of Universidad Nacional de San Luis (San Luis, Argentina) and at the Museo Provincial Carlos Ameghino (Cipolletti, Argentina), using standard methods [81, 82]. To avoid loss of anatomical information, mold and casts of the extracted samples were made and replaced in the original elements [82]. Thin sections were analyzed using a petrographic polarizing microscope (Leica DM 750P). The nomenclature and definitions of structures used in this study are derived from Francillon-Vieillot et al. [83] and de Buffrénil & Quilhac [84].

After a detailed search and examination of the bibliography on the presence of pathologies in non-avian theropods, we have realized a database including all occurrences known so far (see Supplementary Materials 1 and 2). The data base includes taxa, geographical and stratigraphic provenance (Tables S1, S2), possible diagnosis (Tables S3, S4), pathological bone element (Tables S5-S7), and reference utilized. The identification of the elements was based on the bibliography, thus in the cases where the right position is not spelled out is due to the lack of information in the literature. In the cases where we could not identify the right position of axial elements along the series, we differentiated them by “/N”. In the cases where we could not know the amount of the bone with a particular pathology, we considered this group of elements (the word in the plural form) as a single occurrence.

Finally, we have tested the possibility of a significant association among different variables in three different statistical tests using the Chi-square test [85]. The variables utilized in these tests are taxa, type of pathologies, and body regions. In the case of the variables taxa and type of pathologies, we have only taken into account the most sampled categories; for the category taxa we have considered the four categories Tyrannosauridae, Allosauridae, Carcharodontosauridae, and Abelisauridae, while for the category type of pathologies we have considered the three categories: fractures, bite marks, and osteomyelitis. For the category body regions, we have considered the three main body areas: skull, axial, and appendicular. These statistical non-parametric tests were performed using the programs PAST (Paleontological Statistics V4.13) [86] and IBM SPSS (Statistical Package for the Social Sciences V28.0.1). For the tests presented here, we have reported the χ2, p-value, and the degrees of freedom (Table 1). Furthermore, we have reported the occurrences of the most represented pathologies in the most represented taxa (Table S8), the most represented pathologies in the major body regions (Table S12), the major body regions affected by pathologies in the most represented non-avian theropod taxa (Table S16), the residual values (that correspond to positive–negative correlations; Tables S9, S13, S17), the *p*-value of each variable (S10, S14, S18), and the comparison between the observed cases versus expected cases (Tables S11, S15, S19). To reject, or not, the null hypothesis (H0), we have considered a limit value α = 0.05.

**Table 1:** Chi 2 test results after taxa-type of pathologies, body regions-pathologies, and taxa-body regions frequencies comparisons

	**X**^**2**^	**df**	***p*-value**
Taxa-Pathologies	36.274	6	2.44E-06
Body Regions-Pathologies	147.263	4	7.85E-31
Taxa-Body Regions	59.615	6	5.39E-11

*x*^*2*^ Chi-square value, *df* degrees of freedom

## Results and discussion

### Gross morphology

The external morphology of the bones presented here has been described elsewhere [45, 77, 79], thus the following descriptions of the specimens are brief and focused only on abnormal anatomical features. Since *Quilmesaurus* lacks external pathological structure or changings in the cortical portion of the bone, probably due to taphonomic causes, we have only described the caudal vertebrae of *Aucasaurus* and *Elemgasem*.

#### *Aucasaurus garridoi* MCF-PVPH-236 (Fig. 2A-E)

**Fig. 2:**
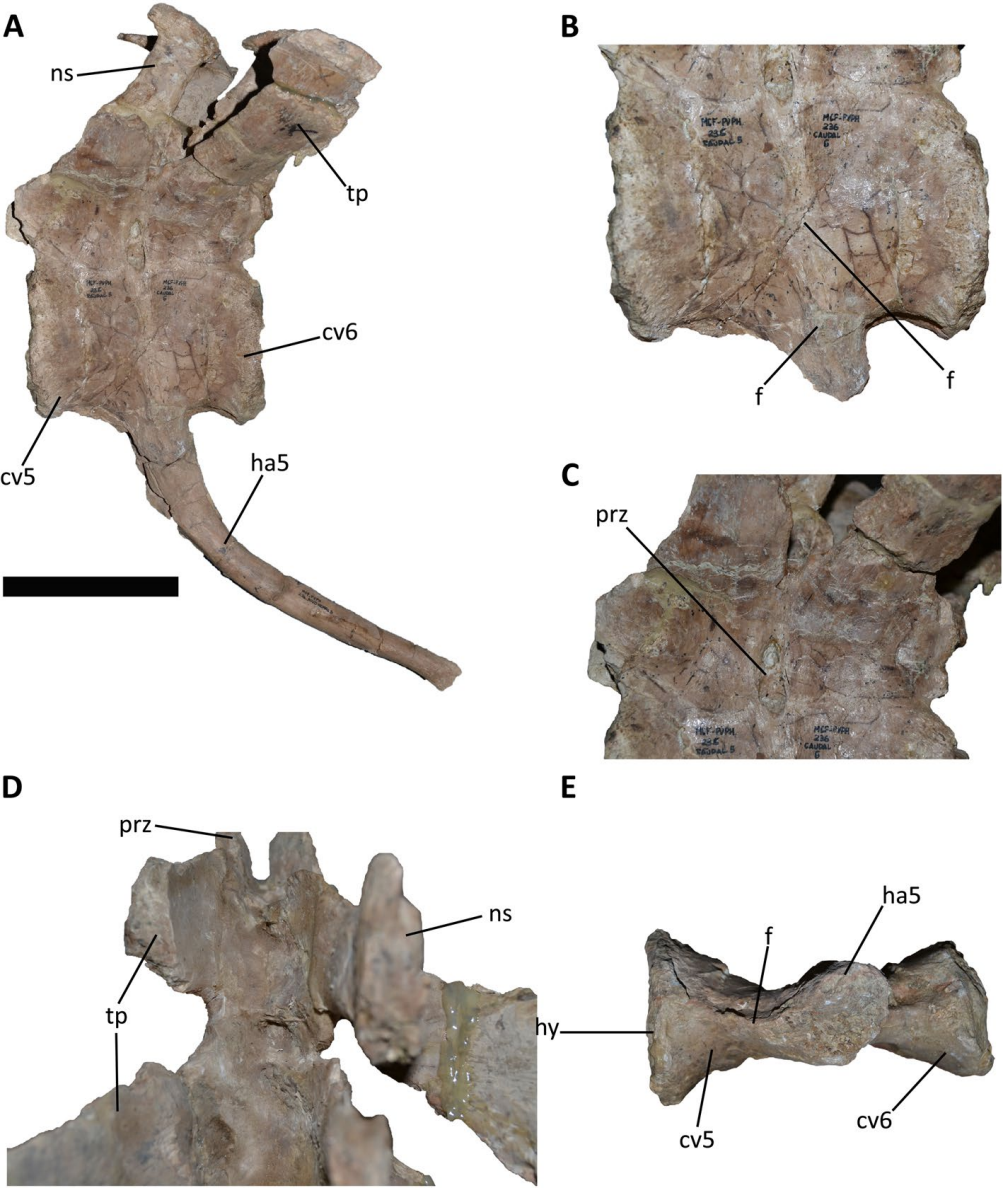
Fifth and sixth caudal vertebra and fifth haemal arch of *Aucasaurus garridoi* MCF-PVPH-236. **A**, the three caudal elements in lateral view; **B**, detail of fused centra and the haemal arch in lateral view; **C**, detail of the partial fusion of the neural arches in lateral view; **D**, detail of the partial fusion of the neural arches in dorsal view; **E**, detail of the fusion among the three elements in ventral view. Abbreviations: cv5, fifth caudal vertebra; cv6, sixth caudal vertebra; f, fusion of articular surfaces; ha5, fifth haemal arch; hs, contact surface for the haemal arch; ns, neural spine; prz, prezygapophysis; tp transverse process. Scale bar only for the image A: 10 cm

The holotype specimen of *Aucasaurus garridoi* MCF-PVPH-236 has a complete caudal series until the thirteenth caudal vertebra, and the corresponding haemal arches. All vertebrae are well-preserved showing low deformation, and the weathering only affected some transverse processes and neural spines. However, the 5th and 6th caudal vertebrae show an atypical condition when compared to the other caudal elements. In fact, these two vertebrae have the centra completely fused with each other, and both are fused to the 5th haemal arch (Fig. 2A). The fusion among these elements is smooth, without signals of irregularity, such as exostosis, osteophytes, or bone lysis (e.g., empty spaces representing fibriscesses [87]) in the cortical bone, except for a faint dorsoventral directed ridge (Fig. 2B). Conversely, the neural arches are partially fused, at least at the level of the zygapophyseal and the hypantrum-hyposphene articulations, whereas the neural spines are unfused (Fig. 2C, D). The centra have an anteroposterior length of 6.5 cm and 6.8 cm respectively, and at the portion where the centra are fused each other (at the posterior articular surface of the 5th and the anterior articular surface of the 6th caudal vertebrae) the transversal width is 3.8 cm. These measurements are the lowest of the *Aucasaurus*’s caudal series [79]. The same condition is observed for the 5th and 6th haemal arches, since they have the lowest lengths (23.9 cm and 17.7 cm respectively) of the *Aucasaurus*’s haemal arch series [79]. The 5th haemal arch is firmly fused to both vertebrae (Fig. 2A, B, E) showing an accentuated posterior bowing (Fig. 2A) respect to the other haemal arches of the series [75, 79].

#### *Elemgasem nubilus* MCF-PVPH-380 (Fig. 3A-C; Fig. 4A-C)

**Fig. 3:**
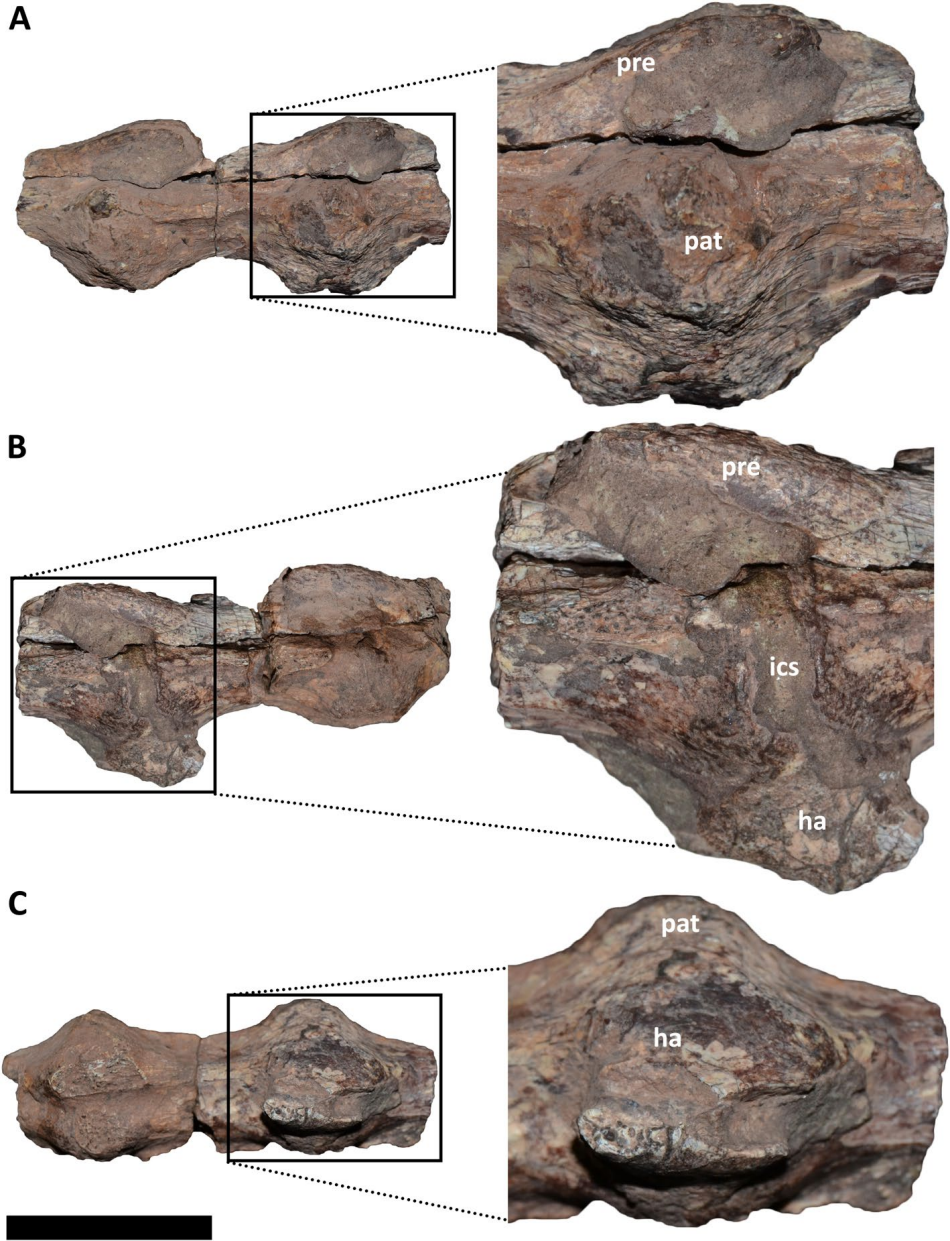
Middle caudal vertebrae of *Elemgasem nubilus* MCF-PVPH-380. In A, lateral right view; B, lateral left view; and C, ventral view. Abbreviations: ha, haemal arch; ics, intercentrum space; pat, pathology; pre, prezygapophysis. Scale bar: 5 cm

**Fig. 4:**
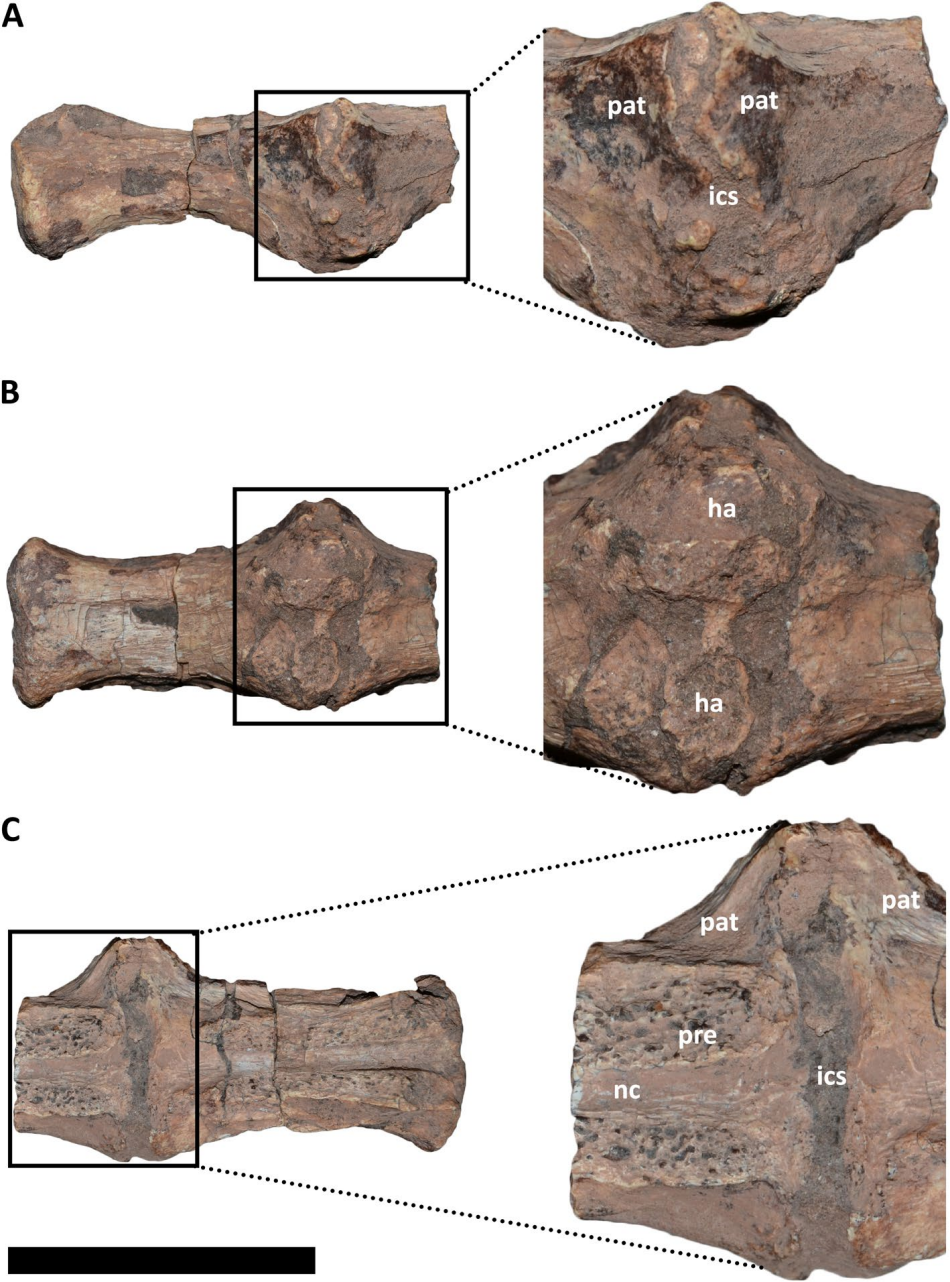
Posterior vertebrae of *Elemgasem nubilus* MCF-PVPH-380. In **A**, lateral right view; **B**, lateral left view; and **C**, ventral view. Abbreviations: ha, haemal arch; ics, intercentrum space; nc, neural canal; pat, pathology; pre, prezygapophysis. Scale bar: 5 cm

The holotype specimen of *Elemgasem nubilus* is represented by several mid and posterior caudal vertebrae [45]. Among them, three vertebrae from the mid-section and two from the posterior section of the tail show a pathological condition (Fig. 3A; Fig. 4A). These vertebrae were found partially articulated, since the middle three ones are fused to one another, and the posterior ones are also fused one another. Furthermore, all vertebrae have the respective haemal arches partially fused (Fig. 3B, C; Fig. 4B). Despite the presence of sedimentary matrix among neural arches, the prezygapophyses, neural spines, and postzygapophyses are discernible, without sign of fusion among them. The mid caudal vertebrae show the neural arch partially separated from the centrum along a fracture, probably due to some taphonomic processes (Fig. 3A), whereas the posterior centra have completely lost the neural arches (Fig. 4A, C). Besides the fusion among the centra, these caudal vertebrae show a pathological condition since there is a bone overgrowth (syndesmophytes [1]), surrounding mainly the right lateral and ventral rims of the centrum articular surfaces (Fig. 3A, C; Fig. 4A-C). These swellings point laterally and have a triangular outline in dorsal/ventral view (Fig. 3C; Fig. 4B, C). The surface in correspondence of the swellings has a quite smooth texture, and the cortical bone lacks cloacae (= channels). It is noteworthy that the pathology is more pronounced on the right side in the middle centra and on the left side in the posterior ones. Moreover, the contact among the middle three vertebrae is almost obliterated on the right side, whereas on the left side is partially visible. For the posterior two centra the articulation is visible in all views, but having an irregular outline, especially in dorsal view where is clearly visible the intercentrum space (Fig. 4C). The ventral portion of the articular surfaces of all centra are articulated and partially fused to the proximal end of the respective haemal arches, which have lost the shafts (Fig. 3B, C; Fig. 4B). Despite the centra and the neural arches have retained their shape and size, thus excluding mechanical trauma, the fusion gives a “bamboo-like” appearance to these vertebrae [1, 74].

### Histological description

#### *Elemgasem nubilus* MCF-PVPH-380

**Fig. 5:**
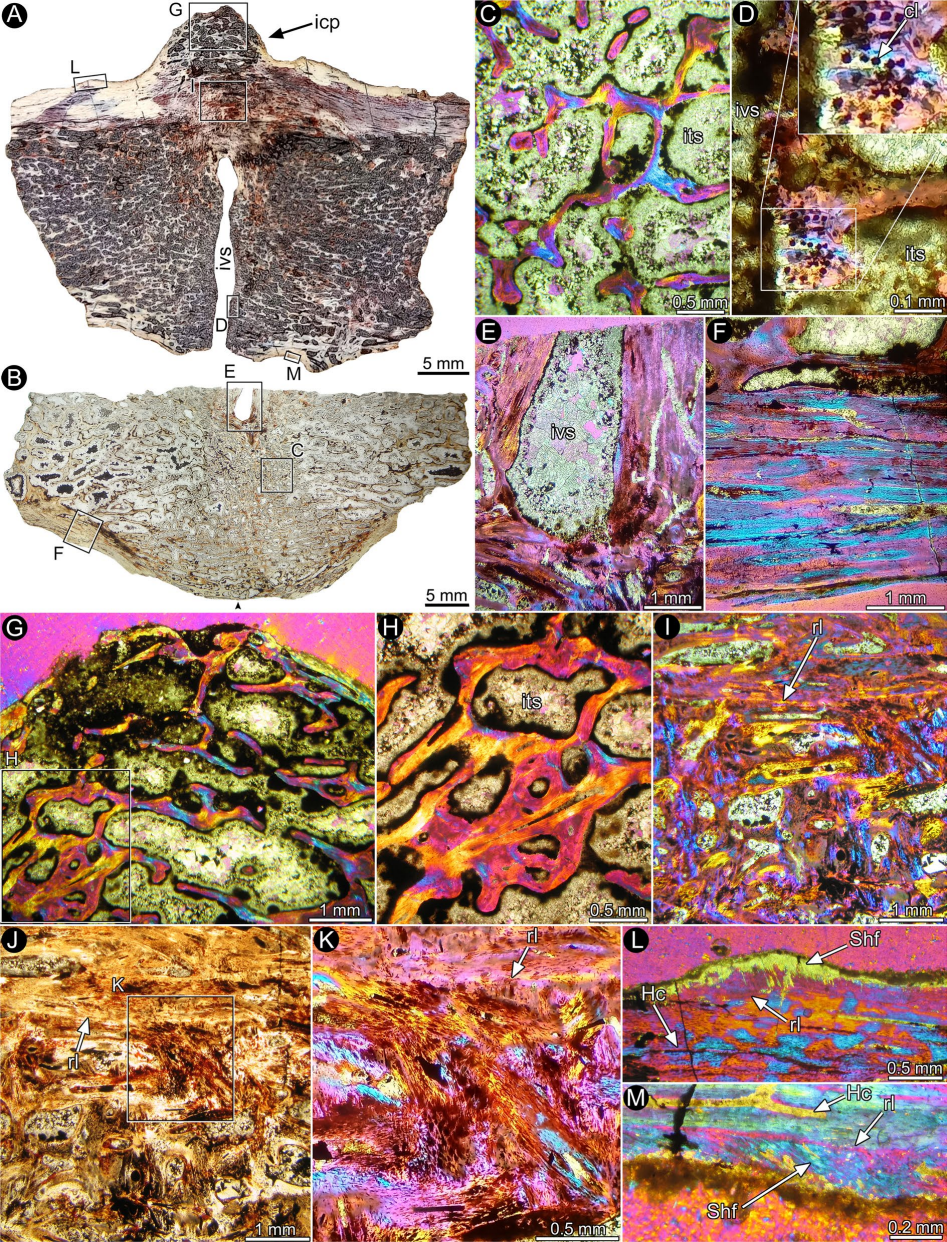
Bone histology of partially fused caudal centra of *Elemgasem nubilus* MCF-PVPH-380. The letters in the inset boxes indicate the position of the detailed pictures in the figure. **A**, **B** Complete longitudinal (**A**) and sagittal (**B**) sections of partially fused centra. The sedimentary matrix has been digitally eroded to for better contrast. The black arrowhead signals the junction area between the centra. **C** Detail of the cancellous bone. **D** Unfused portion of the intervertebral joint showing a preserved layer of calcified cartilage in the articular surface of the centrum. Chondrocyte lacunae are detailed in the enlarged view. **E** Detail of the unfused portion of the intervertebral joint in the sagittal section. **F** Vertebral cortex formed by secondary bone tissue. G, H. General view (**G**) and detail (**H**) of the intercentral protuberance core. Note the prevalence of secondarily formed cancellous bone. **I**-**K** General views (**I**, **J**) and detail (**K**) of the compact bone tissue formed in the intervertebral joint area of the fused vertebral centra. Note the presence of a distinct resorption line dividing two different portions of the compact bone. L, M. Remains of subperiosteal bone showing abundant Sharpey’s fibers. Abbreviations: cl, chondrocyte lacunae; Hc, Haversian canal; icp, intercentral protuberance; its, intertrabecular space; ivs, intervertebral space; rl, resorption line; Shf, Sharpey’s fibers

The centra are mainly occupied by a spongiosa composed of numerous and thin trabeculae and surrounded by a thin outer cortex of compact bone tissue (Fig. 5A, B). The cancellous bone is secondary in origin. Bony trabeculae are composed of secondary lamellar bone formed during remodeling (Fig. 5C).

Both longitudinal and sagittal sections reveal that the centra are not completely fused. In fact, the continuity of the bone tissues between the centra is only evident at the right margin and the ventral region of these elements. Such absence of fusion is not only evidenced by the clear separation between the articular surfaces, which forms a distinct intervertebral joint space of approximately 1.5 mm, but also by the presence of a thin layer of calcified cartilage on these surfaces (Fig. 5D). The sagittal sections reveal that the internal margins of the articular surfaces thicken near the site in which the centra are fused (Fig. 5E). Except for the pathological area, the cortical bone is composed mostly of secondary osteons formed during different generations of remodeling, in which the Haversian canals are mainly oriented in parallel to the centrum main axis (Fig. 5F).

Two well differentiated areas can be distinguished in the regions corresponding to the fused portions of the centra at the right lateral side. The first comprises the core of the intercentral overgrowth and consists of remodeled cancellous bone (Fig. 5G, H). Internal to this area, a distinct region of compact bone tissue is evident (Fig. 5I-K). This portion is formed by abundant secondary osteons, which exhibit two distinct patterns of arrangement. The secondary osteons located adjacent to the cancellous bone of the intercentral overgrowth are roughly arranged with their Haversian canals oriented in parallel to the centrum main axis. Conversely, the secondary osteons formed toward the core of the vertebrae exhibit a strong variation with regard to their orientation. Such heterogeneity results in an irregular microstructural pattern, with a chaotic arrangement of the Haversian canals (Fig. 5K). These two areas of secondary compact bone are clearly bounded by a distinct resorption line. Remains of primary cortical bone preserved in the ventral and lateral sides of the centra exhibits a poorly vascularized tissue which contain abundant extrinsic (i.e. Sharpey’s) fibers (Fig. 5L, M).

The sagittal section reveals that both centra are mostly well fused (Fig. 5B). In this regard, there is a clear continuity of the cancellous bone between these two bone elements. It is worth noting that intertrabecular spaces tend to be more reduced in the former articulation area of the centra.

#### *Quilmesaurus curriei* MPCA-PV-100

**Fig. 6:**
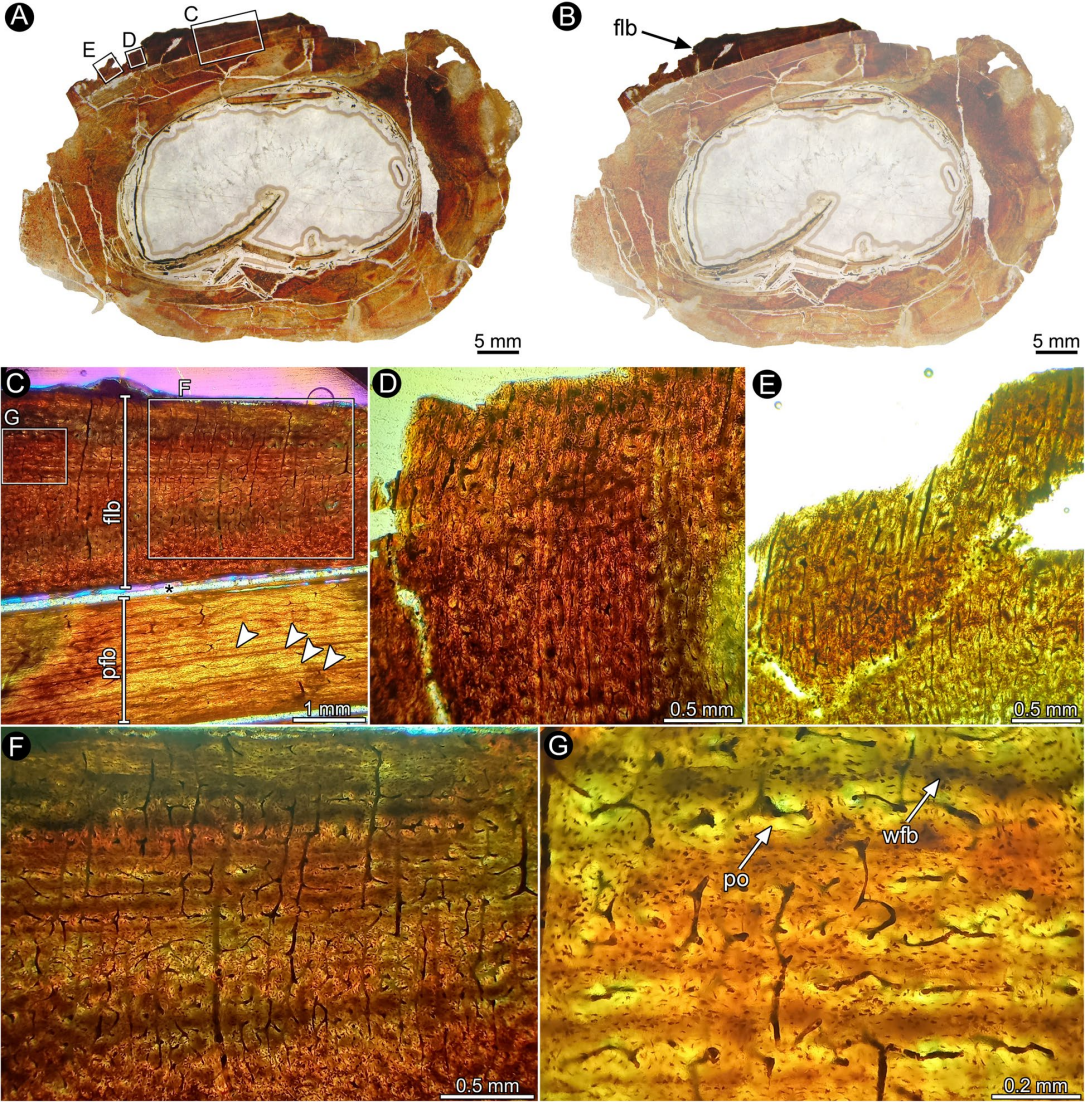
Bone histology of the right tibia of *Quilmesaurus curriei* MPCA-PV-100. The letters in the inset boxes indicate the position of the detailed pictures in the figure. **A**, **B** Complete section of the element. Note the presence of a distinct layer of highly vascularized fibrolamellar bone. The opacity of the medullary cavity and most of the bone tissue has been digitally reduced for better contrast. **C** General view of the cortex showing cortical bone formed by parallel fibered bone, interrupted by lines of arrested growth (white arrowheads), and fibrolamellar bone. Note that the cortex is broken in the boundary between these two different types of bone tissues (asterisk). **D**-**F** General views of the fibrolamellar bone. Detailed view of the fibrolamellar bone. Abbreviations: flb, fibrolamellar bone; pfb, parallel fibered bone; po, primary osteon; wfb, woven fibered bone

Although a detailed histological description of the specimen will be published elsewhere (Cerda et al. in prep), here we provide a general characterization of the sample. The cross section of the tibia exhibits a well vascularized cortex of compact bone that encircles a free medullary cavity (Fig. 6A, B). Primary bone tissue, mostly formed by parallel fibred bone tissue, predominates in most of the compacta (Fig. 6B). Cyclical growth marks are distinct from the perimedullary to the outer cortex. The histological features of the compact bone exhibit a noticeable variation at the lateral portion of the outer cortex. The subperiosteal cortex in this area exhibits a distinct layer of highly vascularized fibrolamellar bone tissue that reaches a maximum thickness of 3.7 mm, lacking any kind of growth marks (Fig. 6C-F). The fibrolamellar bone is formed by a matrix of woven fibered bone tissue, in which osteocyte lacunae are densely packed and haphazardly distributed (Fig. 6G). The latter feature strongly contrasts with the parallel fibered bone that predominates in the compacta, where the osteocyte lacunae exhibit an elongated shape and are roughly concentrically arranged to the shaft main axis. Radially and longitudinally oriented vascular canals (organized as primary osteons) predominate. Obliquely oriented canals are also abundant and they commonly anastomose and form patches of reticular bone. The change of the fibrolamellar and the parallel fibered bone formed below is abrupt, signed by weak lines that have induced the breakage of the cortex in the boundary between these two highly differentiated tissues. This portion of fibrolamellar bone lacks cyclical growth marks, which indicates that the same was deposited within a single cycle of growth.

### Differential diagnosis

Despite the majority of the study on the diseases in extinct organisms is based on gross morphological description, and in some cases preferred to other methodologies [88], in the last years the paleohistology and the computed tomography have been implemented to know the internal change of the bone structure when affected by pathology [48, 51, 89]. These approaches are helpful methodologies to study maladies especially when some of them show similar external appearance whereas the internal arrangement of the bone responds in different ways [23], or when diseases do not leave any external evidence [90, 91]. Therefore, the knowledge of the microstructural anatomy, plus the observations of the external structures, gives us a more precise diagnosis about the disease than when it is only taken in account the macroscopic details, removing possible misidentifications [23]. As to the cases presented here, the CT-scan and the paleohistology have allowed us to recognize, with a high degree of confidence, the possible pathology suffered by MCF-PVPH-236, MCF-PVPH-380, and MPCA-PV-100. The maladies present in these abelisaurid specimens extends the paleopathological record for the group Ceratosauria, since reported diseases in this group were restricted to few specimens of the abelisaurid *Majungasaurus crenatissimus* mostly due to traumatic injuries [24, 26].

#### *Aucasaurus garridoi* (MCF-PVPH-236)

**Fig. 7:**
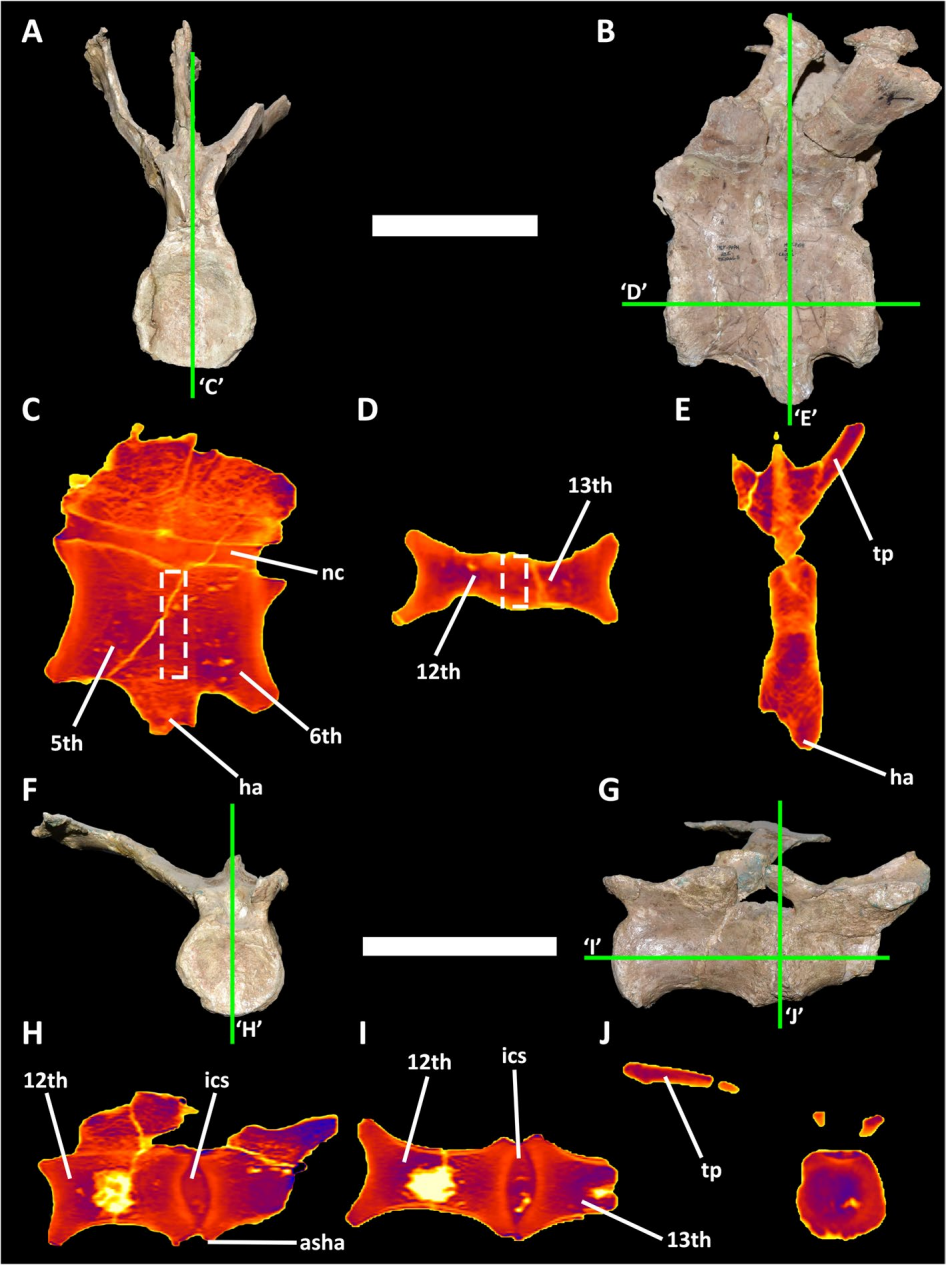
CT-scans of pathologic and normal caudal vertebrae of *Aucasaurus garridoi* MCF-PVPH-236. **A**, anterior; and **B**, lateral views of the 5th and 6th caudal vertebrae, and 5th haemal arch. Tomographic images in **C**, parasagittal section; **D**, coronal section; and **E**, transverse section. **F**, anterior; and **G**, lateral views of the 12th and 13th caudal vertebrae. Tomographic images in **H**, parasagittal section; **I**, coronal section; and **J**, transverse section. The dashed-line boxes indicate the zone among fused vertebrae without an intercentrum space. The green lines indicate the planes of the slices. Abbreviations: 5th, fifth caudal vertebra; 6th, sixth caudal vertebra; 12th, twelfth caudal vertebra; 13, thirteenth, caudal vertebra; asha, articulation surface for the haemal arch; ha, haemal arch; ics, intercentrum space; nc, neural canal; tp, transverse process. Scale bars 10 cm

The pathological condition observed in MCF-PVPH-236 shows common traits observed in other maladies, but distinctive characteristics allow to discern it from any other disease. A malignant tumor is inconsistent with the condition present in MCF-PVPH-236 since it produces overgrowths or lytic lesions [58, 74, 92], which are absent in this specimen. The Paget’s disease is, in some cases, represented by vertebral fusion where the intercentrum space is reduced or absent [93], as in MCF-PVPH-236. However, the specimens that show this pathology present centra that have maintained the whole size and have an osseous bump on the side of the articular surfaces [93], which is absent in MCF-PVPH-236. Also the spondyloarthropathy and diffuse idiopathic skeletal hyperostosis (DISH) produce vertebral ossification; however, in both cases the centra are perfectly developed and the calcification involves only the rim of the articular surfaces [1, 94]. The lack of irregular exostosis, and the presence of more posterior vertebrae discard the truncation of a portion of the tail [14, 26]. Taking into account the above-mentioned considerations, we consider that the following traits observed in MCF-PVPH-236 fits with a congenital disorder, possibly related with block vertebrae (Fig. 7A-E):

1. Fused centra and neural arches;
2. lacking intercentrum space;
3. lacking exostosis, osteophytes, and cloacae;
4. centra anteroposteriorly and mediolaterally reduced than the remnant elements.

Congenital disorders of the vertebral column, such as failure of formation (wedge vertebra or hemivertebra) or defect of segmentation (fused centra) [95, 96], have been documented in several extinct [12, 35, 36, 97–104] and extant organism [105–109]. This malformation possibly is due to genetic defects that produce disruption of the normal somite (vertebral elements precursors) formation and/or segmentation [96, 108], or caused by environmental factors during embryogenesis, such as hypoxia, high temperature, or high level of carbon monoxide [101, 107, 108].

Congenital disorders that affect the axial skeleton block vertebrae were poorly noticed in non-avian dinosaurs [36, 110]. Newman [111] mentioned two dorsal vertebrae that have the centra completely fused for a specimen of *Tyrannosaurus rex* (AMNH 5027) that posteriorly were considered, along with the last cervical and the first dorsal of the same specimen, as block vertebrae [110]. Despite vertebral centra are anterioposteriorly shorter than the other ones ([112]; plate. XXVII), a feature of congenital block vertebrae present also in MCF-PVPH-236 (Fig. 7A, D, F, I), the Molnar’s statement [110] is based on the external fusion of the dorsal vertebrae of the specimen AMNH 5027. However, Rothschild & Molnar [34] observed the absence of intervertebral space, thus indicating a complete fusion of both centra (through radiological examination), and supporting a failure of segmentation between these vertebral elements. The CT scanning of the 5th and 6th caudal vertebrae (Fig. 7A, B) of MCF-PVPH-236 shows fused centra also lacking spacing between them (Fig. 7C, D), a pathognomonic condition in the cases of block vertebrae [95]. The bone density in the middle portion (in correspondence of the dorsoventral ridge and where supposedly the centra contact) is only slightly denser than the remaining centra (Fig. 7C), as observed in the *Apatosaurus* (cf. *A. ajax*) specimen WDC LA-188 [36]. Moreover, the density of the proximal portion of the 5th haemal arch is almost indistinguishable from the centra, lacking clear articular surfaces among them (Fig. 7C, E). Therefore, the total union without signs of articulations implies an unfinished development of these caudal elements. In contrast, non-pathologic vertebrae of MCF-PVPH-236, as the 12th and 13th caudal vertebrae (Fig. 7F, G), show a well-defined intercentrum space filled by sediment, a different bone density between the articular surfaces and the remaining centra, and a differentiated articulation surfaces for the haemal arch (which was found unfused to them) (Fig. 7H-J). The presence of congenital malformations in caudal vertebrae were mentioned also for a specimen of *Allosaurus fragilis* and the holotype of *Poekilopleuron bucklandii* [99], though detailed macroscopic and microstructural studies on these specimens are needed to confirm this diagnostic. Hence, MCF-PVPH-236 is among the few well-documented occurrences for this type of congenital vertebral abnormality [34, 36] and, at the same time, the first case among non-tetanuran theropods. Furthermore, the congenital malformation of the MCF-PVPH-236 affected the tail portion, as observed in other non-avian dinosaur specimens [34, 36] but different from other extinct tetrapod where the affected portion were the neck and the trunk [35, 97, 100, 101, 110].

Interestingly, Molnar [110] concluded that due to the presence of similar responses to vertebral development dysfunctions in mammals and archosaurs (block vertebrae are recovered also in humans [113]), some developmental processes have remained practically unaltered since the common ancestor of archosaurs and mammals. The case reported here supports this scenario, where congenital deformations appeared early within Tetrapoda [97, 100, 101], without modifications of the developmental processes during the ontogeny of several internal lineages [110].

#### *Elemgasem nubilus* (MCF-PVPH-380)

The fusion among articular surfaces of neural arches and/or vertebral centra may be the physiological response of multiple pathologies [1]. However, the proliferation of new bone forming outgrowths reduces the range of possible diseases. The presence of well-developed centra that maintain their size and shape and the presence of an intercentrum space excludes a congenital malformation [34, 96]. Paget’s disease of bone is also ruled out since in this case the intercentrum space is reduced or absent [93]. A bone fracture due to traumatic injury is healed with new bone generation forming an overgrowth commonly called”callus”; however, MCF-PVPH-380 lacks fractures or internal disruption (fracture line) and the overall axes of the vertebrae are unaffected maintaining their general shape. Moreover, vertebrae with traumatic injuries are barely documented in dinosaurs [73], with only a few cases mentioned in the literature [5, 8, 12, 14, 26, 53, 114]. The absence of typical traits of osteomyelitis, such as cloacae, drainage canals or sinus, filigree texture and irregular architecture of the cortical surface in correspondence of the outgrowths [1, 26, 38, 51, 70, 115, 116], rules out an infection as cause of the bone proliferation in MCF-PVPH-380. Scheuermann’s disease is also rejected due to the absence of wedge-shaped centra and the absence of localized erosion caused by subchondral cyst at synovial vertebral joint [67, 117], in the articular surface and a ventral narrowing of the vertebral centra. A degenerative disease of the vertebral column is the osteochondrosis intervertebralis; however, the osteophytes, the typical structure observed in this pathology, are absent in MCF-PVPH-380. We also reject the presence of Hypervitaminosis A since in the latter the enthesial ossifications are characterized by apposition of periosteal “laminar” bone [118]. We rule out DISH, a condition that is characterized by calcification and ossification of ligaments and entheses (ligament and tendon insertion sites) [1], since the bone outgrowths in MCF-PVPH-380 lack the pathognomic dripped candle wax appearance. Moreover, DISH appears in senile individuals and the calcification is separate from the centra producing bridgings externally to the joint capsule [1, 119, 120], whereas MCF-PVPH-380 supposedly died during a sub-adult ontogenetic stage [45] and the newly formed bone is deposited within the annulus fibrosus. The fusion among vertebrae and haemal arches is a trait poorly documented in DISH occurrences, but it is more common in specimens where spondyloarthropathy was reported [12]. Thus we consider that the following pathological traits present in MCF-PVPH-380 are the consequence of spondyloarthropathy:

1. Presence of unaltered vertebral centra with exostosis located on the anterior and posterior rim of the articular surfaces of the centra;
2. The exostosis, as to result of a pronounced ankylosis, was generated within the joint capsule, producing the “bamboo” appearance of the vertebrae;
3. Among the affected vertebrae the intercentrum space is perfectly preserved.

Among extant vertebrates, spondyloarthropathy is the major non-traumatic osseous pathology at least in lizards and crocodylians [121]. However, the occurrence of spondyloarthropathy in the fossil record is limited to a few specimens of tetrapod vertebrates, precluding an accurate evaluation of its frequency. Among non-avian tetrapods, this disease was recorded in *Dimetrodon*, *Ctenorhachis*, *Diadectes*, *Lunaophis*, *Shringasaurus*, and an indeterminate phytosaur [94, 122–124]. The record of spondyloarthropathy among non-avian dinosaurs is also limited to few cases since it was reported in ceratopsids, hadrosaurs, and sauropodomorphs [33, 57, 125, 126], and only one occurrence among non-avian theropods [74]. Thus, the presence of this pathology in MCF-PVPH-380 is the first evidence for a non-tetanuran theropod, and the second one for a non-avian theropod.

#### *Quilmesaurus curriei* (MPCA-PV-100)

The particular bone tissue observed in MPCA-PV-100 has been mostly recovered in appendicular bones of several amniotes, with poor occurrences in axial bones [71, 127]. In fact, the presence of the radial fibrolamelar bone (RFB) has been reported in a radius of an indeterminate gorgonopsian [128], in a femur of the titanosuchid *Jonkeria parva* [129], possibly in a humerus referred to the phytosaur *Smilosuchus gregorii* [130], and in long bones of several dinosaurs [31, 62, 69, 71, 90, 91, 127, 131–136]. In some cases, the RFB was found along with a bone tissue similar to the medullary bone (reproductive tissue present in female birds [137]), with consequence reconsideration of the latter as a tissue response of the egg laying, thus used as a calcium reservoir (e.g. [131], but see [138]). The main feature that characterizes the RFB is an unexpected change of the vascularization that in this type of fast-formed tissue is predominantly directed radially and perpendicularly to the circumference of the bone wall [91, 127, 131, 133]. Other traits that distinguish the RFB are the form, distribution, and the amount of the osteocyte lacunae. As to MPCA-PV-100, the periosteal portion with RFB has randomly oriented osteocyte lacunae with globular shape; whereas, the underlying parallel fibred bone tissue shows flattened osteocyte lacunae distributed concentrically. An abrupt change of the shape and/or the density between the osteocyte lacunae of the RFB and the surrounding bone tissue is always present in all cases where this type of bone tissue was reported [62, 69, 71, 90, 91, 134]. However, it seems the RFB shows different grades of the radial vascularization, with an increase of radial canals among different specimens [127]. As to MPCA-PV-100, it has a condition resembling a specimen of *Stegosaurus* or a Triassic neotheropod dinosaur [90, 134], but with a lower radial vascularization than some sauropodomorph specimens or *Psittacosaurus mongoliensis* [71, 127, 133].

The lack of fracture, callus, or puncture marks in MPCA-PV-100 rules out an infection (e.g., osteomyelitis), traumatic injury or some type of stress force as cause of the abrupt change in the cortical bone [60, 62, 129]. At the same time, we reject the possibility of muscle insertion migration related with a shift from facultative bipedality to quadrupedality (which could be the cause of the presence and the orientation of the RFB tissue in some ornithischian dinosaurs [132, 133]), since theropod dinosaurs are obligate bipeds [139, 140]. However, we cannot refute that the presence of the RFB in MPCA-PV-100 tibia is the outcome of overstrain due to a weakened (i.e., that has suffered a trauma) fibula such as observed in two *Maiasaura* specimens [132], since this bone is unknown in *Quilmesaurus*. Notably, Chinsamy & Tumarkin-Deratzian [131] argued about the possibility of osteopetrosis or hypertrophic osteopathy as the cause of the presence of the RFB in a non-avian dinosaur bone, although these authors regarded osteopetrosis more reliable since that specimen also shows a medullary-like bone deposited endosteally (see also [134]). Conversely, Chinsamy & Tumarkin-Deratzian [131] regarded the hypertrophic osteopathy as possible cause of several occurrence of the RFB in the fossil record when this tissue is only restricted to the periosteal portion [127, 133, 135], as well as it is observed in MPCA-PV-100.

Nowadays, we do not know which pathology has produced the formation of this bone tissue, and at the same time other possible physiological producers are not discarded. Whether the condition of the tibia of MPCA-PV-100 will confirm as a pathology, it will be among the few non-avian theropod occurrences where a disease has affected a weight-bearing bone [130]. For instance, *Sanjuansaurus*, a basal theropod, present the right distal tibia with a pathological bone outgrowth [141], whereas a neotheropod fibula from the Upper Triassic of New Mexico has a callus on the diaphysis [134]. Other non-avian theropod specimens with pathological weight-bearing bones are restricted to *Allosaurus*, *Gorgosaurus*, *Albertosaurus*, and *Tyrannosaurus* [110, 142].

### Pathology occurrences in non-avian theropod fossil record

From its first definition and apparition in the literature [143–146] paleopathology has gradually occupied an increasingly important role in paleontology, providing a useful tool to understand some ecological and biological aspects of the ancient faunae [1, 88]. As regards non-avian dinosaurs, each year new specimens affected by some type of pathology are discovered and published, improving our knowledge about the diversity of diseases present in the past and in which bone they had proliferated [147]. Among dinosaurs, the first pathological evidence was recognized in the tetanuran theropod *Poikilopleuron* [148]; nowadays, after 185 years, a large number of non-avian theropod specimens have preserved evidence of some type of maladies.

In our study we have found in the literature more than 337 bone affected by pathology, although in some cases there are more than one pathological bone per specimen (e.g., FMNH PR 2081; FMNH PR 2836; SMA 0005; Supplementary Materials 1 and 2), or, in the other case, for some specimens we could not discern the exact number of the pathological bones, thus we considered them as a single occurrence. Due to latter consideration, we regard that the amount of the cases collected in the literature could be a sub-estimation of the actual number of pathologies known in the theropod fossil record.

**Fig. 8:**
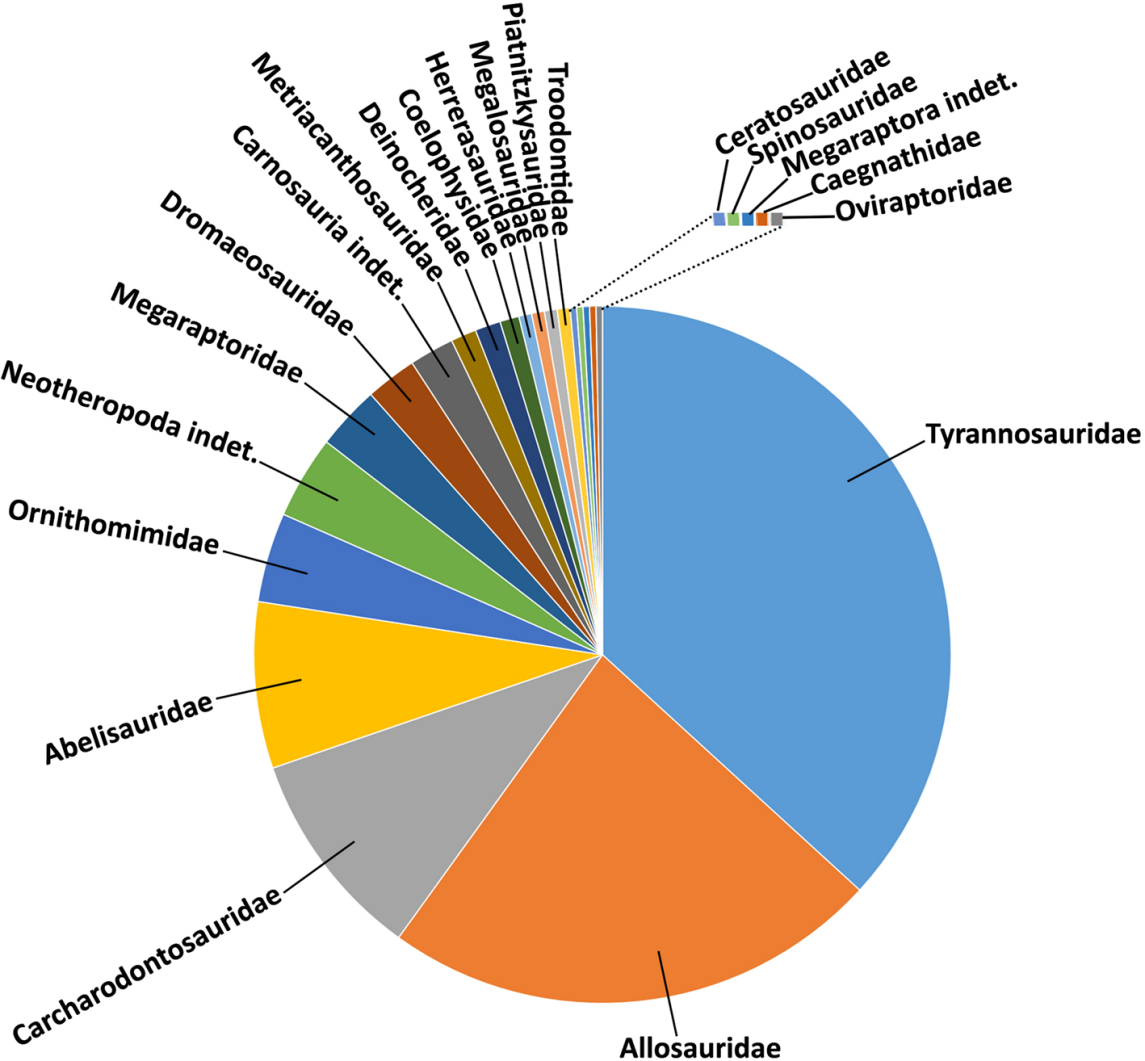
Graph showing the distribution of diseases among non-avian theropods (percentages are listed in the Supplementary Materials 1 and 2)

We found pathological evidences in 39 genera (plus several indeterminate specimens) within 18 families (e.g., Abelisauridae, Allosauridae, Dromaeosauridae), in 5 higher groups (e.g., Ceratosauria, Carnosauria, Coelurosauria) (Supplementary Materials 1 and 2). The most represented family is Tyrannosauridae with almost 37% of total occurrences, followed by Allosauridae with almost 23%, Carcharodontosauridae with 9.8%, and Abelisauridae with almost 8%. The remaining families have an incidence lower than the 5%, in some cases represented by a single occurrence (Fig. 8; Supplementary Materials 1 and 2). These results are probably the consequence of two main bias; 1), the most represented groups are those include medium to large theropods, and it is expected since large sized individuals have more possibility to fossilize than small ones [149–151]; and, 2) medium and large theropod specimens are represented by larger samples (e.g., *Allosaurus*, *Albertosaurus*, *Mapusaurus*, *Tyrannosaurus*; [28, 47, 152, 153]) than the small ones. These two factors have conditioned the vertebrate paleontologists to focus on the presence of pathologies mainly in large well-preserved specimens such as *Allosaurus*, *Majungasaurus*, and *Tyrannosaurus* [2, 24, 26, 73, 142].

**Fig. 9:**
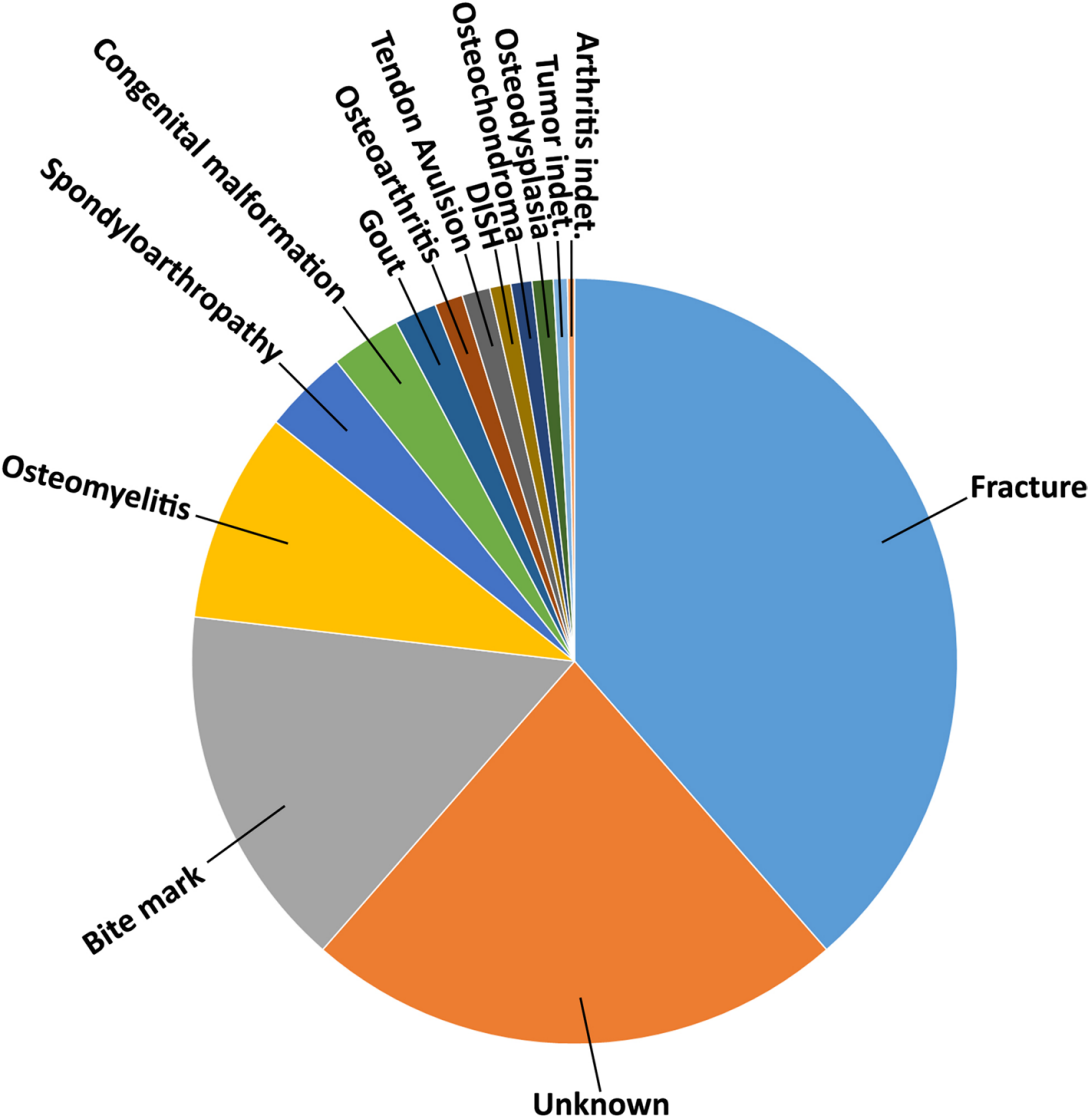
Graph showing the types and the amount of diseases recorded in non-avian theropods (percentages are listed in the Supplementary Materials 1 and 2)

Considering the diversity of disease occurrences, fractures (healed or not) are the most recorded pathologies so far (Fig. 9; Supplementary Materials 1 and 2). In fact, almost 39% of the whole data set presents some type of fracture, including 18 cases with pseudarthrosis. This value reflects partially previous results on extinct non-avian theropods [2, 73] and living theropod dinosaurs (birds) in which disorders after traumatic events are the most represented diseases [154]. There is a high number, 22.8% of the sample, of cases where it was impossible to diagnose a specific pathology or it was simply omitted in the original publication. However, several authors have proposed possible alternatives in the cases where they were unable to establish a single cause [26, 47, 73]. Bite marks are well-represented since more than 15% of all bones show puncture wounds due to bites.

Osteomyelitis (we considered the cases where this was the only pathology identified) is recorded in almost 9% of the sample, which indicates that infections are rather common, at least among some non-avian theropod groups [116]. This value is slightly higher if we consider the cases where osteomyelitis was associated with other diseases, such as fractures and bite marks, reaching 15% of the sample. However, infectious cases in extant birds have a higher incidence when compared to extinct non-avian dinosaurs, approaching 30% of the whole studied sample [116]. Fractures, bite marks, and osteomyelitis are the unique pathologies present in all three skeleton principal divisions (cranial, axial, and appendicular). Spondyloarthropathy is a pathology characterized by reactivation of bone formation, ossification at sites of tendon, ligament, or capsule insertion, with peripheral joint erosion or/and fusion of distinct elements [1]. It is recovered in 3.5% of the sample, and only in *Allosaurus fragilis* and *Elemgasem nubilus* [45, 74]. However, spondyloarthropathy is the most represented pathology along the caudal portion, an outcome in accordance with its record among sauropod dinosaurs [54]. The presence of congenital malformations is reported in almost 3% of the sample, being *Aucasaurus* the oldest record among non-avian theropods with this pathology so far. Gout is a type of arthritis resulting from the deposition of uric acid [1, 155], and it was recognized only among a few coelurosaurs with an incidence of 1.7%. Osteoarthritis, perhaps an atrophic or degenerative process that affects the cartilaginous portion of the joints with production of osteophytes [1], is present in 1.1% of the sample (only recovered in *Allosaurus fragilis*). With the same percentage, tendon avulsion was recognized in several tetanurans appendicular elements. DISH, a possible senile phenomenon characterized by ossification of ligaments [1], was found in only three occurrences of two genera, *Majungasaurus* and *Tyrannosaurus* [26, 34]. However, several specimens of *Allosaurus* and some caudal elements of the holotype of *Neovenator salerii* could be affected by DISH. Osteodysplasia, a genetic disease that produces a slowing, disordering, or accelerating of the growth of the bone [156], was discerned only in the forelimb of *Dilophosaurus wetherilli* [58] with a total incidence of 0.89%. Tumors were identified in few specimens, as the benign bone tumor osteochondroma recognized in some tyrannosaurids [34, 110, 157] with an incidence of 0.89%, and indeterminate tumors present in *Allosaurus* and *Dilophosaurus* [58, 74] with an incidence of 0.59%. These values are similar to the rates of tumors in extant birds and crocodylians [116]. Finally, an indeterminate arthritis was recovered only in a single specimen (0.29%).

**Fig. 10:**
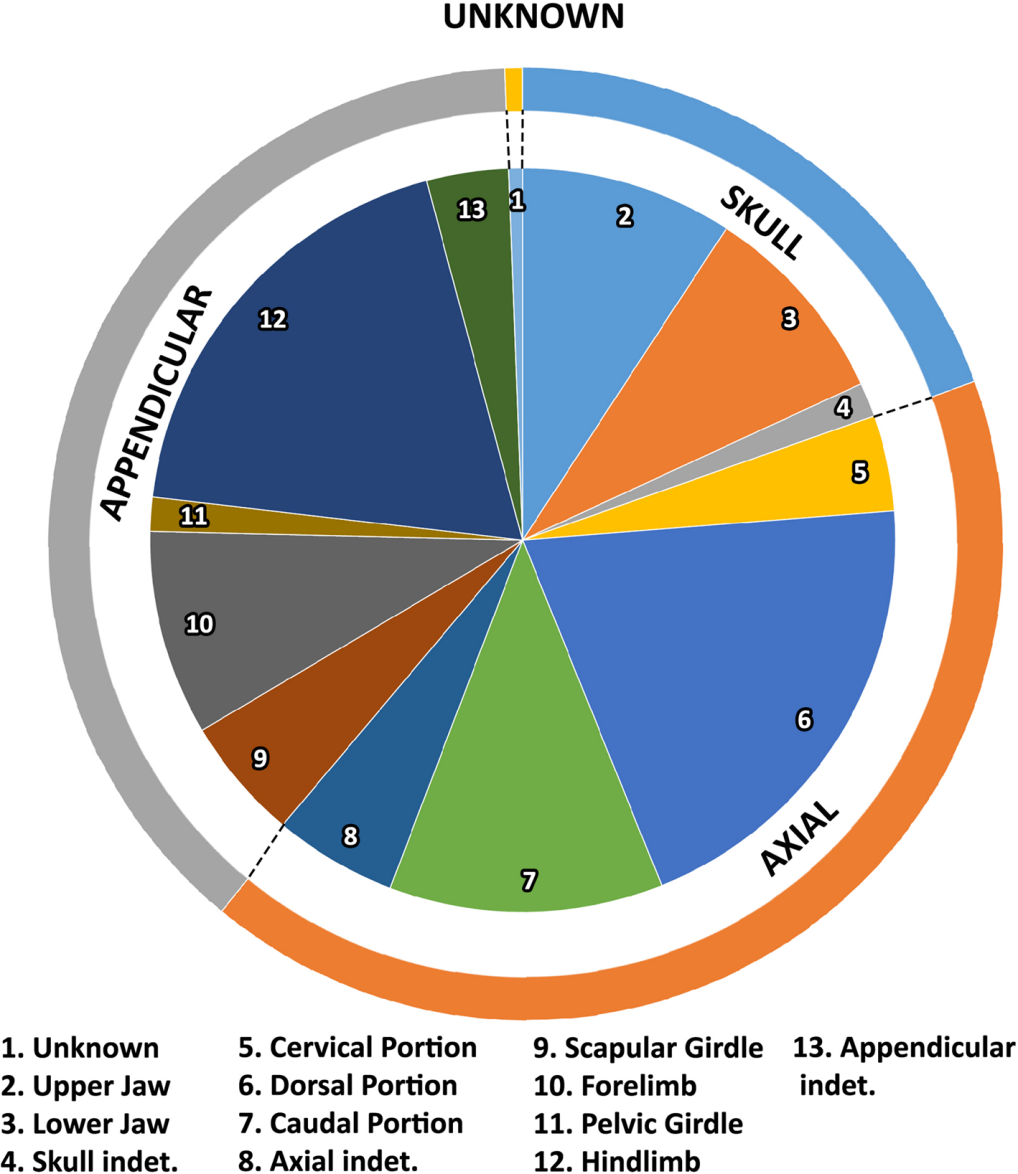
Graph showing the distribution of diseases among the different portions of the skeleton in non-avian theropods (percentages are listed in the Supplementary Materials 1 and 2)

Considering the major divisions of the skeleton (Fig. 10; Supplementary Materials 1 and 2), the most affected portion is the axial skeleton with almost 42% of occurrences, followed by the appendicular with 38%, and the cranial with 19.5%. The remaining 0.5% are unidentified bones. Perhaps these results reflect the higher number of elements that compose the axial skeleton in comparison with cranial and appendicular portions, and the presence of some maladies that were mainly diagnosticated in the axial portion (e.g., congenital deformation, spondyloarthropathy, DISH). Among the subareas of the principal regions (Fig. 10; Supplementary Materials 1 and 2), the dorsal portion of the axial skeleton is the most represented with 20% of the incidences, hindlimbs 19%, caudal portion 11.8%, upper and lower jaws, and forelimb each with approximately 9%. The remaining categories are represented approximately among the 5.3% and the 0.6%. To date, no sacral vertebrae have been found with pathology in non-avian theropods, thus potentially congruent, at least for trauma injuries, with the hidden position among ilia and the partial or total fusion of these bones in some groups (e.g., Ceratosauria). These results are biased by the high occurrences of fractures recorded in the dorsal portion (Supplementary Materials 1 and 2), while body parts covered by deep muscular packages (e.g., stylopodium, cervical portion) are more protected from traumatic injuries [110]. Furthermore, some elements are poorly represented in the literature as the weight-bearing bones tibia and femur (Supplementary Materials 1 and 2), possibly due to their higher resistance to mechanical injuries. Serious pathology in weight-bearing bones of a bipedal animal would hamper the feeding activities or would yield it to be an easy target for predators [110, 158].

### Behavioural insights from theropod paleopathologies

The number of pathologies recorded in among different lineages of theropods can potentially provide insights on injuries provoked by the frequency of certain behaviours. Results of the Chi-square test found several significant associations that results informative in this regard (Table 1), especially for comparisons between the frequency of different types of pathologies for certain clades. In particular, we found a positive association between Tyrannosauridae and bite marks, Allosauridae and fractures, and Abelisauridae and osteomyelitis (Table S9 in Supplementary Material 1), whereas we found a negative association between Tyrannosauridae and fractures, and between Allosauridae and bite marks. When we compared the variables body regions and type of pathologies, the Chi-square test also yielded a strong significant association (Table 1). In this case, we have found a positive association between skull and bite marks and between axial and appendicular skeleton and fractures, whereas we found a negative association between skull and fractures and between axial and appendicular skeleton and bite marks (Table S13 in Supplementary Material 1). When we considered the variables taxa and body regions, we also found a strong significant association (Table 1), corresponding to positive association in the cases of Tyrannosauridae and skull, Allosauridae and appendicular skeleton, Carcharodontosauridae and Abelisauridae and axial skeleton. Conversely, a negative association was found for Tyrannosauridae and axial skeleton, Allosauridae and skull, Carcharodontosauridae and Abelisauridae and appendicular skeleton (Table S17 in Supplementary Material 1). The statistical tests of these associations between clades with pathologies and injured skeletal regions may be explained by the frequency of specific behaviours in different theropod clades and/or different physiological responses of the skeleton to different pathologies.

The clearest example of this likely is the positive association between tyrannosaurids and bite marks, recovered in skull bones rather than in other parts of the skeleton. Such a strong association is suggestive of the presence of antagonistic behaviours in these theropods, including aggressive and highly energetic combats that could occur in intra- or interspecific interactions and be related to feeding, courtship ⁄ mating, territoriality, or dominance [6, 11, 16, 27, 116]. Conversely, the negative correlation of bite marks present in the skull of allosaurids suggest the lack of frequent biting in the cephalic regions among their behavioural repertoire. On the other hand, a positive association among allosaurids and fractures (mainly found in the appendicular skeleton) supports an active predatory lifestyle [8, 73] that could have commonly caused fractures in the axial and appendicular skeleton, as evidenced by the high number of ribs, gastralia, chevrons, fibulae, and phalanges with healed fractures.

It is worth highlighting that the occurrences of bite marks assigned to tyrannosaurids in our database are only those that show clear signs pre-mortem infliction and subsequent healing, such as healed tissue or infection-induced structures like osteomyelitis on cranial and post-cranial bones (e.g. [6, 16, 17, 27, 28, 157, 159, 160]). These remodelling signs confirm the pre-mortem origin of the marks, suggesting that the affected individuals survived the biting events, which likely resulted from either intra- or interspecific interactions. Moreover, we have taken into account mainly the bite marks recorded in skull bones, which are considered as ‘low economy’ elements due to their lesser nutritional value (e.g. [161]). This parallels observed behaviors in both modern and ancient crocodyliforms, which sometimes target these less valued parts [162] of their prey/opponent, some of which survive the attacks [163]. Marks on bones that display healing structures and occurring on elements considered as the last choice in terms of food supply (e.g., skull bones), are here considered as pathological and not as the results of taphonomic processes. Several studies mentioned the presence of bite marks on theropod bones, especially in tyrannosaurids and allosaurids [161, 164], but in many of these the marks do not have signs of healing processes. We have excluded these occurrences from our database, as these could represent post-mortem damage and fall within a taphonomic rather than pathological context.

The positive correlation between osteomyelitis and Abelisauridae (principally in the skull and axial skeleton) shows this disease was frequent in this group of Ceratosaurian theropods but not in the other clades tested (all of which belong to the large clade Tetanurae). The positive correlation may indicate a physiological tendency to this type of infections in ceratosaurs that contrasts with the unusual occurrence of this infections in large-sized theropod tetanurans (Supplementary Materials 1 and 2; see also [73]).

## Conclusions

Paleopathology has improved our knowledge about some biological and ecological aspects of ancient life through the study of diseases in extinct animals. This consideration is particularly evident for non-avian dinosaurs since studies focused on paleopathologies in these organisms have grown exponentially in the last decade, giving us information about physiological response and indirect information to infer possible behaviours associated with certain type of injuries. Our knowledge about pathologies in the fossil record has also improved after the application of new methodologies, such as histology and computed tomography, that have provided us the possibility to explore the internal structure of pathologic bones.

**Fig. 11:**
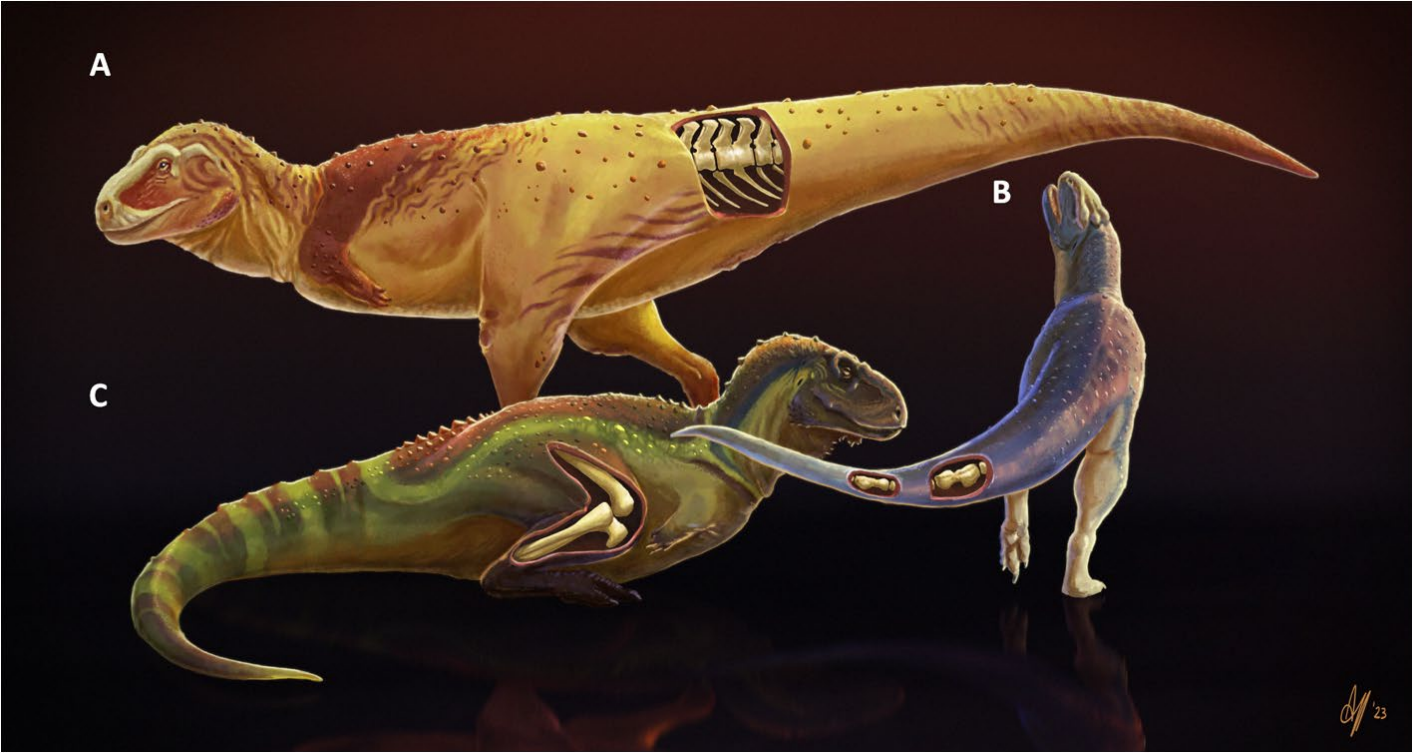
Pathological abelisaurid specimens. **A**, *Aucasaurus garridoi* MCF-PVPH-236 (congenital malformation in anterior caudal vertebrae); **B**, *Elemgasem nubilus* MCF-PVPH-380 (spondyloarthropathy in middle and posterior caudal elements); and **C**, *Quilmesaurus curriei* MPCA-PV-100 (possible pathology in the right tibia). Artwork by Alessio Ciaffi

Here we have analyzed the holotype specimens of the abelisaurid *Aucasaurus*, *Elemgasem*, and *Quilmesaurus*, that show a pathological condition (Fig. 11). For a more accurate diagnosis of the diseases, we have carried out histological analyses and computed tomography, in addition to their macroscopical description. *Aucasaurus* has the 5th and 6th caudal vertebrae and the 5th haemal arch firmly fused to each other, lacking evidence of bone lysis due to infection or bone formation (e.g., osteophytes, callus, etc.) due to traumatic injuries, metabolic, or neoplastic diseases. The external morphology and the CT-scans show that these caudal elements were not able to finalize the development and to separate each other. We consider this pathology as a congenital malformation, the first occurrence among non-tetanuran theropods. *Elemgasem* has three the middle and two posterior caudal vertebrae and the respectively haemal arches (only the proximal portion) totally or partially fused. Moreover, there is a lateral bone overgrowth on the right rims of the centrum articular surfaces. The histological analysis shows that this overgrowth is not separated by the cortical bone, and there is an evident intercentrum space among the centra. This pathology is here considered as spondyloraptropathy, the first case among non-tetanuran theropods and the second occurrence among non-avian theropods. The condition observed in *Quilmesaurus* was the more arduous to discern its cause, since it lacks any external evidence of pathologies. Moreover, the radial fibrolamellar bone that characterizes the tibia of *Quilmesaurus* was previously considered as a consequence of different diseases or as a physiological response to external forces. At the moment, we cannot discard any possible diseases or other causes.

This constitutes the first study focused on paleopathologies for the clade Brachyrostra and the third one for the clade Abelisauridae in general, and the first occurrences of pathologies in non-tetanuran theropods from South America. With this work, we have improved the knowledge about paleopathology in abelisaurid theropods and in dinosaurs in general.

We also presented here an exhaustive review of the literature and assembled a database with all pathological occurrences in non-avian theropods. This compilation has brought several outcomes, the foremost ones are: 1) most of pathologies are recovered among large-sized theropods; 2) traumatic and infectious disease are the most represented pathologies; 3) the portion of the skeleton most commonly affected are the appendicular and axial regions. Furthermore, statistical tests suggest tyrannosaurid had frequent bite marks in the cephalic region, which likely resulted from intraspecific antagonistic behaviour whereas allosaurids injuries in the ribs and hindlimb may reflect their active predatory lifestyle. Finally, abelisaurids show a tendency to develop osteomyelitis that seem to be unusual in large-bodied tetanuran theropods.

### Supplementary Information

**Additional file 1:** **Table S1.** Occurences arranged according to the pathological specimens. Different colors indicate different taxa. **Table S2.** Percentage of each taxon with respet to the whole sample. **Table S3.** Occurences arranged according to the type of pathology. Different colors indicate different pathologies. **Table S4.** Percentage of each pathology with respet to the whole sample. **Table S5.** Occurences arranged according to the pathological regions of the skeleton. Different colors indicate different body regions. **Table S6.** Percentage for each major body region affected by a pathologcial condition with respet to the whole sample. **Table S7.** Percentage of each secondary body region affected by a pathological condition with respet to the whole sample. **Table S8.** Occurences of the most represented pathologies in the most represented non-avian theropod taxa. **Table S9.** Ajusted residuals after Chi-square test. **Table S10.** *P*-values. *In yellow significant *p*-values. **Table S11.** Observed cases versus expected cases after Chi-square test. **Table S12.** Occurences of the most represented pathologies in the major body regions. **Table S13.** Ajusted residuals after Chi-square test. **Table S14.** *P*-values. *In yellow significant *p*-values. **Table S15.** Observed cases versus expected cases after Chi-square test. **Table S16.** Occurences of major body regions affected by pathological conditions in the most represented non-avian theropod taxa. **Table S17.** Ajusted residuals after Chi-square test. **Table S18.** *P*-values. *In yellow significant *p*-values. **Table S19.** Observed cases versus expected cases after Chi-square test.

**Additional file 2. **

## Data Availability

The fossil specimens examined here are housed in the paleontological collections of the Museo Carmen Funes (Plaza Huincul, Argentina) and Museo Provincial Carlos Ameghino (Cipolletti, Argentina). All data generated for this study is provided in the manuscript and in the files named Supplementary Materials 1 and 2.
