# Supplementary material for: New information on paleopathologies in non-avian theropod dinosaurs: a case study on South American abelisaurids
Source: BMC Ecol Evol. 2024 Jan 31;24:6. doi: 10.1186/s12862-023-02187-x (PMC10829224; doi:10.1186/s12862-023-02187-x)
Supplement: Supplementary file 2 — Additional file 2. [file 12862_2023_2187_MOESM2_ESM.docx]

**SUPPLEMENTARY REFERENCES**

Anné, J., Garwood, R. J., Lowe, T., Withers, P. J., & Manning, P. L. (2015). Interpreting pathologies in extant and extinct archosaurs using micro-CT. *PeerJ*, *3*, e1130.

Archer, B., & Babiarz, J. P. (1992). Another tyrannosaurid dinosaur from the Cretaceous of northwest New Mexico. Journal of Paleontology, 66(4), 690-691.

Baur, G. 1890. A review of the charges against the Paieontological Department of the U. S. Geological Survey, and of the defence made by Prof. O. C. Marsh. American Naturalist 24:298-304.

Bell, P. R. (2010). Palaeopathological changes in a population of *Albertosaurus sarcophagus* from the Upper Cretaceous Horseshoe Canyon Formation of Alberta, Canada. *Canadian Journal of Earth Sciences*, *47*(9), 1263-1268.

Bell, P. R., & Coria, R. A. (2013). Palaeopathological survey of a population of *Mapusaurus* (Theropoda: Carcharodontosauridae) from the late cretaceous Huincul formation, Argentina. *PloS one*, *8*(5), e63409.

Bell, P. R., Currie, P. J., & Lee, Y. N. (2012). Tyrannosaur feeding traces on Deinocheirus (Theropoda:? Ornithomimosauria) remains from the Nemegt Formation (Late Cretaceous), Mongolia. Cretaceous Research, 37, 186-190.

Bell, P. R., Rothschild, B. M., & Tanke, D. H. (2011). First report of gout in an ornithomimid (Dinosauria: Therpopda) from the horseshoe canyon formation, Alberta. New Mexico Museum of Natural History and Science Bulletin, 53, 166–168.

Brochu, C. A. (2003). Osteology of *Tyrannosaurus rex*: insights from a nearly complete skeleton and high-resolution computed tomographic analysis of the skull. *Journal of Vertebrate Paleontology*, *22*(sup4), 1-138.

Brusatte, S., Benson, R. B., & Hutt, S. (2008). The osteology of *Neovenator salerii* (Dinosauria: Theropoda) from the Wealden group (Barremian) of the Isle of Wight. *Monograph of the Palaeontographical Society*, *162*, 1-75.

Calvo, J. O., Porfiri, J. D., Veralli, C., Novas, F., & Poblete, F. (2004). Phylogenetic status of *Megaraptor* *namunhuaiquii* Novas based on a new specimen from Neuquén, Patagonia, Argentina. *Ameghiniana*, *41*(4), 565-575.

Carpenter, K. 1982. Baby dinosaurs from the Late Cretaceous Lance and Hell Creek formations and a description of a new species of theropod. Contributions to Geology (University of Wyoming) 20: 123-134.

Carpenter, K., and Smith, NI. 2001. Forelimb osteologv and biomechanics of *Tyrannosaurus rex*. P.90-116 In: Tanke DH, Carpenter K, Skrepnick MW (eds.). Mesozoic vertebrate life: new research inspired by the paleontology of Philip J. Currie. Indiana Universitv Press, Bloomington.

Chinzorig, T., Beguesse, K. A., Canoville, A., Phillips, G., & Zanno, L. E. (2022). Chronic fracture and osteomyelitis in a large‐bodied ornithomimosaur with implications for the identification of unusual endosteal bone in the fossil record. The Anatomical Record.

Clark JM, Norell MA, Chiappe LM. 1999 An oviraptorid skeleton from the Late Cretaceous of Ukhaa Tolgod, Mongolia, preserved in an avianlike brooding position over an oviraptorid nest. Am Mus. Novit. 3265, 1–36.

Coria, R. A., & Currie, P. J. (2016). A new megaraptoran dinosaur (Dinosauria, Theropoda, Megaraptoridae) from the Late Cretaceous of Patagonia. PLoS One, 11(7), e0157973.

Currie, P. J., & Zhao, X. J. (1993). A new carnosaur (Dinosauria, Theropoda) from the Jurassic of Xinjiang, People's Republic of China. *Canadian Journal of Earth Sciences*, *30*(10), 2037-2081.

D’Anastasio, R., & Capasso, L. (2004). Post-microtraumatic cervical osteoarthritis in a cretaceous dinosaur. *Reumatismo*, *56*(2), 124-128.

Farke, A. A., & O'Connor, P. M. (2007). Pathology in *Majungasaurus crenatissimus* (Theropoda: abelisauridae) from the Late Cretaceous of Madagascar. *Journal of Vertebrate Paleontology*, *27*(S2), 180-184.

Foth, C., Evers, S. W., Pabst, B., Mateus, O., Flisch, A., Patthey, M., & Rauhut, O. W. (2015). New insights into the lifestyle of *Allosaurus* (Dinosauria: Theropoda) based on another specimen with multiple pathologies. *PeerJ*, *3*, e940.

Gilmore, C. W. 1920. Osteology of the carnivorous Dinosauria in the United States National Museum, with special reference to the genera *Antrodemus* (*Allosaurus*) and *Ceratosaurus*. Bulletin of the United States National Museum 110: 1-159.

Glut, D. F. 2000. Dinosaurs: The Encyclopedia, Supplement 1. Jefferson, N.C.: McFarland

Griffin, C. T. (2018). Pathological bone tissue in a Late Triassic neotheropod fibula, with implications for the interpretation of medullary bone. *New Jersey State Museum Investigations*, *6*, 2-10.

Gutherz, S. B., Groenke, J. R., Sertich, J. J., Burch, S. H., & O'Connor, P. M. (2020). Paleopathology in a nearly complete skeleton of *Majungasaurus crenatissimus* (Theropoda: Abelisauridae). *Cretaceous Research*, *115*, 104553.

Hamm, C. A., Hampe, O., Schwarz, D., Witzmann, F., Makovicky, P. J., Brochu, C. A., Reiter, R., & Asbach, P. (2020). A comprehensive diagnostic approach combining phylogenetic disease bracketing and CT imaging reveals osteomyelitis in a Tyrannosaurus rex. *Scientific reports*, *10*(1), 18897.

Hanna, R. R. (2002). Multiple injury and infection in a sub-adult theropod dinosaur *Allosaurus fragilis* with comparisons to allosaur pathology in the Cleveland-Lloyd Dinosaur Quarry collection. *Journal of Vertebrate Paleontology*, *22*(1), 76-90.

Harris, J. D. (1998). *A Reanalysis of Acrocanthosaurus atokensis, its Phylogenetic Status, and Paleobiogeographic Implications, Based on a New Specimen from Texas: Bulletin 13* (Vol. 13). New Mexico Museum of Natural History and Science.

Hone, D. W. E., & Tanke, D. H. (2015). Pre-and postmortem tyrannosaurid bite marks on the remains of Daspletosaurus (Tyrannosaurinae: Theropoda) from Dinosaur Provincial Park, Alberta, Canada. *PeerJ*, *3*, e885.

Jacobsen, A. R., Tanke, D. H., & Carpenter, K. (2001). Tooth-marked small theropod bone: an extremely rare trace. In: Tanke DH, Carpenter K, (eds.). Mesozoic vertebrate life: new research inspired by the paleontology of Philip J. Currie. Indiana University Press, Bloomington, Indiana:, 58-63.

Lambe, L. M. 1917. The Cretaceous theropodous dinosaur *Gorgosaurus*. Geological Suruey of Canada, Memoirs 100: 1-84.

Lee, A. H., & Werning, S. (2008). Sexual maturity in growing dinosaurs does not fit reptilian growth models. Proceedings of the National Academy of Sciences, 105(2), 582-587.

Lipkin, C., & Carpenter, K. (2008). Looking again at the forelimb of Tyrannosaurus rex. Tyrannosaurus rex: the tyrant king, 166-190.

Madsen, J.H., J. I976. A second new theropod dinosaur from the Late Jurassic of east central Utah. Utah Geology 3: 51-60.

Marsh, A. D., & Rowe, T. B. (2020). A comprehensive anatomical and phylogenetic evaluation of Dilophosaurus wetherilli (Dinosauria, Theropoda) with descriptions of new specimens from the Kayenta Formation of northern Arizona. *Journal of Paleontology*, *94*(S78), 1-103.

McGowan, C. 1991. Dinosaurs, Spitfires, and Sea Dragons. Cambridge,

Molnar, R. E. 1991. The cranial morphology of *Tyrannosaurus rex*. Palaeontographica A, 217: 137 -176.

Molnar RE (2001) Theropod paleopathology: a literature survey. In: Tanke DH, Carpenter K, Skrepnick MW (eds.). Mesozoic vertebrate life: new research inspired by the paleontology of Philip J. Currie. Indiana University Press, Bloomington, Indiana: 337–363.

Moodie, R. L. 1923. Paleopathology. Urbana: University of Illinois Press.

Newman, B. 1970. Stance and gait in the flesh-eating dinosaur *Tyrannosaurus*. Biological Journal of the Linnean Society 2: 119-123.

Norell, M. A., and P. J. Makovicky. 1997.lmportant features of the dromaeosaur skeleton: information from a new specimen. American Museum Novitates 3215: 1-28.

Norell, M. A., E. S. Gaffney, and L. Dingus. 1995. Discovering Dinosaurs in the American Museum of Natural History. New York: Knopf.

Ostrom, J. H. 1976. On a new specimen of the Lower Cretaceous theropod dinosaur *Deinonychus antirrhopus*. Breviora 439: 1,-21.

Parks,'W. A. 1928. *Albertosaurus arctunguis*, a new species of theropodous dinosaur from the Edmonton Formation of Alberta. Uniuersity of Toronto Studies, Geological ser.,25: 1-42.

Petersen, K., Isakson, J. I., & Madsen Jr, J. H. (1972). Preliminary study of paleopathologies in the Cleveland-Lloyd dinosaur collection. In *Utah Academy Proceedings* (Vol. 49, No. 1, p. 44-47).

Peterson, J. E., Henderson, M. D., Scherer, R. P., & Vittore, C. P. (2009). Face biting on a juvenile tyrannosaurid and behavioral implications. Palaios, 24(11), 780-784.

Reid, R. E. H. 1996. Bone histology of the Cleveland-Lloyd dinosaurs and of dinosaurs in general. Part 1: Introduction to bone tissues. Brigham Young Uniuersity Geology Studies 41 25-71.

Rothschild, B.M. 1997. Dinosaurian paleopathology. In: J.O. Farlow and M.K. Brett-Surman (eds.), The Complete Dinosaur, 426–448. Indiana University Press, Bloomington.

Rothschild, B., & Lambert, H. W. (2021). First documentation of a greenstick fracture in the fossil record. Possible gout also noted in *Arkansaurus fridayii*. Historical Biology, 33(9), 1349 1351.

Rothschild BM, & Molnar RE. 2008 Tyrannosaurid pathologies as clues to nature and nurture in the Cretaceous. In *Tyrannosaurus rex*, the tyrant king (eds PL Larson, K Carpenter), pp. 286–306. Bloomington, IN: Indiana University Press.

Rothschild BM, Tanke DH. 2005. Theropod paleopathology. In: Carpenter K, ed. *The carnivorous dinosaurs*. Bloomington: Indiana University Press. 351-365.

Rothschild, B., O'Connor, J., & Lozado, M. C. (2022). Closer examination does not support infection as cause for enigmatic *Tyrannosaurus rex* mandibular pathologies. Cretaceous Research, 140, 105353.

Rothschild, B. M., D. H. Tanke, and K. Carpenter. 1997. *Tyrannosaurs* suffered from gout. Nature 387: 357-358.

Rothschild, B. M., Tanke, D. H., & Ford, T. L. (2001). Theropod stress fractures and tendon avulsions as a clue to activity. In: Tanke DH, Carpenter K, Skrepnick MW (eds.). Mesozoic vertebrate life: new research inspired by the paleontology of Philip J. Currie*. Indiana University Press, Bloomington*, 331-336.

Russell, D. A. 1970. *Tyrannosaurs* from the Late Cretaceous of western Canada. National Museum of Natural History, Publicdtions in Palaeontology 1:1-34.

Samathi, A., Weluwanarak, J., Duanyai, P., Kaikaew, S., & Suteethorn, S. (2023). An unusual metatarsal of theropod dinosaur from the lower cretaceous of Thailand: the first detailed study of paleopathology in Megaraptora. Historical Biology, 1-6.

Schweitzer, M. H., Wittmeyer, J. L., & Horner, J. R. (2005). Gender-specific reproductive tissue in ratites and *Tyrannosaurus rex*. Science, 308(5727), 1456-1460.

Senter, P., & Juengst, S. L. (2016). Record-breaking pain: the largest number and variety of forelimb bone maladies in a theropod dinosaur. *PLoS One*, *11*(2), e0149140.

Sereno, P. C., & Novas, F. E. (1994). The skull and neck of the basal theropod Herrerasaurus ischigualastensis. *Journal of Vertebrate Paleontology*, *13*(4), 451-476.

Stovall, J. W., & Langston, W. (1950). *Acrocanthosaurus atokensis*, a new genus and species of Lower Cretaceous Theropoda from Oklahoma. *The American Midland Naturalist*, *43*(3), 696-728.

Sullivan, R. M., Tanke, D. H., & Rothschild, B. M. (2000). An impact fracture in an ornithomimid (Ornithomimosauria: Dinosauria) metatarsal from the Upper Cretaceous (Late Campanian) of New Mexico. Dinasours of New Mexico: Albuquerque, New Mexico, USA, New Mexico Museum of Natural History and Science Bulletin, 17, 109-111.

Tanke, D. H., and P. J. Currie. 1995. Intraspecific fighting behavior inferred from toothmark trauma of skulls and teeth of large carnosaurs (Dinosauria). Journal ofVertebrate Paleontology 15 (suppl. to no. 3): 55A. (Abstract.)

Tanke DH, Currie PJ. 1998 Head-biting behavior in theropod dinosaurs: paleopathological evidence. Gaia 15, 167–184.

Tanke, D. H., & Rothschild, B. M. (2002). Dinosores: an annotated bibliography of dinosaur paleopathology and related topics—1838–2001. *Bull. N.M. Mus. Nat. Hist.* 20:1–96

Tsogtbaatar, C., Cullen, T., Phillips, G., Rolke, R., & Zanno, L. E. (2022). Large-bodied ornithomimosaurs inhabited Appalachia during the Late Cretaceous of North America. Plos one, 17(10), e0266648.

'Websteq D. 1999 . A dinosaur named Sue. National Geographic Magazine 195 (6):46-59.

Welles, S. P. (1984). Dilophosaurus wetherilli (Dinosauria, Theropoda). Osteology and comparisons. Palaeontographica Abteilung A, 85-180.

Wilkin, J.T.R. (2019) Review of pathologies on MOR 693: An Allosaurus from the Late Jurassic of Wyoming and implications for understanding allosaur immune systems. PaleorXiv. <http://doi:10.31233/osf.io/f3rh6>.

Williamson, T. E., and T. D. Carr. 1999. A new tyrannosaurid (Dinosauria: Theropoda) partial skeleton from the Upper Cretaceous Kirtiand Formation, San Juan Basin, New Mexico. New Mexico Geology 2l:42- 43. (Abstract.)

Xing, L., Bell, P. R., Rothschild, B. M., Ran, H., Zhang, J., Dong, Z., ... & Currie, P. J. (2013). Tooth loss and alveolar remodeling in Sinosaurus triassicus (Dinosauria: Theropoda) from the Lower Jurassic strata of the Lufeng Basin, China. Chinese Science Bulletin, 58, 1931-1935.

XING, L. D., DONG, H., PENG, G. Z., SHU, C. K., HU, X. D., & JIANG, H. (2009). A scapular facture in Yangchuanosaurus hepingensis (Dinosauria: Theropoda). Geological Bulletin of China, 28(10), 1390-1395.

Xing, L., Rothschild, B. M., Du, C., Wang, D., Wen, K., & Su, J. (2022). New palaeopathology cases of *Allosaurus fragilis* (Dinosauria: Theropoda). *Historical Biology*, 1-6.

Zhao, X. J., & Currie, P. J. (1993). A large crested theropod from the Jurassic of Xinjiang, People's Republic of China. *Canadian Journal of Earth Sciences*, *30*(10), 2027-2036.
